# Changing health related quality of life and outcomes in heart failure by age, sex and subtype

**DOI:** 10.1016/j.eclinm.2023.102217

**Published:** 2023-09-14

**Authors:** Claire A. Lawson, Lina Benson, Iain Squire, Francesco Zaccardi, Mohammad Ali, Simon Hand, Umesh Kadam, Wan Ting Tay, Ulf Dahlstrom, Lars H. Lund, Gianluigi Savarese, Carolyn S.P. Lam, Kamlesh Khunti, Anna Strömberg

**Affiliations:** a Department of Cardiovascular Sciences, University of Leicester, United Kingdom; b NIHR Leicester Biomedical Research Centre –Cardiology, Department of Cardiovascular Sciences, College of Life Sciences, University of Leicester, Leicestershire, United Kingdom; c Karolinska Institutet, Stockholm, Sweden; d Leicester Real World Evidence Unit, University of Leicester, Leicestershire, United Kingdom; e Diabetes Centre, University of Leicester, Leicestershire, United Kingdom; f National Institute for Health Research (NIHR) Applied Research Collaboration - East Midlands (ARC-EM), University of Leicester, Leicestershire, United Kingdom; g Health Sciences, University of Leicester, Leicestershire, United Kingdom; h Heart Centre Singapore, Singapore; i Department of Cardiology and Department of Health, Medicine and Caring Sciences, Linkoping University, Linkoping, Sweden; j Duke-National University of Singapore, Singapore; k Department of Health, Medicine and Caring Sciences, and Department of Cardiology, Linkoping University, Sweden

**Keywords:** Heart failure, Health, Hospitalization, Mortality, Prognosis

## Abstract

**Background:**

There are calls to integrate serial recordings of health related quality of life (HRQoL) into routine care, clinical trials and prognosis. Little is known about the relationship between change in HRQoL and outcomes in heart failure (HF) patients by age, sex and HF subtype.

**Method:**

From the Swedish Heart Failure Registry (SwedeHF; 2008–2019), patients were categorised by reduced (<40%, HFrEF), mildly-reduced (40–49%, HFmrEF) and preserved (≥50%, HFpEF) ejection fraction. HRQoL was measured using Euro-QoL-5D visual analogue scale (EQ5D-vas), collected at baseline and 1-year. Baseline EQ5D-vas scores were categorised by: “best” (76–100), “good” (51–75), “bad” (26–50), and “worst” (0–25). Change in EQ5D-vas was categorised as ‘no significant change’ (<5 points increase/decrease); some worsening (5–9 points decrease); considerable worsening (≥10 points decrease); some improvement (5–9 points increase); considerable improvement (≥10 points increase). Associations with admission and death were estimated and interactions with patient sub-groups tested.

**Findings:**

Among 23,553 patients (median age 74 [66–81] years, 8000 [34%] female), baseline EQ5D-vas was worse in older patients, women, and those with HFpEF compared to their respective counterparts. Compared to patients with the “best” EQ5D-vas, the adjusted associations for admission for those with “good”, “bad” and “worst” EQ5D-vas were, respectively: HR 1.09 (1.04, 1.14), 1.27 (1.21, 1.33) and 1.39 (1.28, 1.51). Compared to no significant change in EQ5D-vas, the adjusted estimates for admission following some improvement, considerable improvement, some worsening and considerable worsening were, respectively: HR 0.91 (0.82, 1.01), 0.75 (0.70, 0.81), 1.04 (0.92, 1.16) and 1.25 (1.16, 1.35). Results were similar amongst groups and for HF admission and death.

**Interpretation:**

Change in HRQoL was an independent indicator of risk of admission and death in people with all HF subtypes, irrespective of age and sex.

**Funding:**

10.13039/501100000272NIHR.

**Research in context**

**Evidence before this study**

There have been recent calls to integrate serial recordings of health related quality of life (HRQoL) into routine care, clinical trials and prognosis. In Heart failure (HF), baseline HRQoL is closely aligned with physiological parameters and physician assessed clinical status and is known to influence outcomes. Less is known about changes in HRQoL over time and whilst HRQoL varies among different groups with HF, prior studies have been conducted mostly at an aggregate level. Little is known about the role of changes in HRQoL over time, among patients with HF according to age, sex and HF subtype.

**Added value of this study**

This is the largest study to examine change in health related quality of life (HRQoL) by sex, age and HF subtype in >23,000 patients with heart failure. We found that baseline HRQoL differed considerably among people with HF, with worse HRQoL in women, older adults and patients with HFpEF. Despite these differences, HRQoL and its change over a year, were significant and consistent predictors of hospitalization and death, regardless of sex, age and HF-subtype.

**Implications of all the available evidence**

Serial recordings of HRQoL in routine care provide a simple, cost effective and patient-centred approach to prognosis and care planning in people with HF.

## Introduction

Heart failure (HF) affects an estimated 56 million people worldwide and more than 9 million people in Europe.^1^ Diagnosis of HF is associated with higher hospitalization rates than other chronic conditions^2^ and has a 30% mortality rate during the first year.^3^ People with HF often experience episodic symptoms of congestion, functional deterioration and poor health related quality of life (HRQoL).^4^, ^5^, ^6^ Monitoring clinical status is therefore critical to identify deterioration and to plan treatment and care.

Clinical status in HF is measured using a number of different approaches including biomarkers, echocardiographic and electrocardiographic parameters, clinical signs, symptoms reported by patients and HRQoL. HRQoL is a multidimensional concept that can be assessed using validated questionnaires to give a global score and which is closely aligned with physiological parameters,^7^ symptom change^8^ and change in physician assessed clinical status.^9^ Recently there have been calls to integrate serial recordings of HRQoL into routine care for the purposes of quality assessment^10^ and into interventional trials, as an important patient related outcome.^11^ HRQoL measures are a non-invasive, cost effective and patient-centred approach to monitoring clinical change and responsiveness to clinical treatments. Furthermore, evidence over two decades has shown baseline HRQoL to be associated with cardiovascular^12^, ^13^, ^14^, ^15^, ^16^ and all-cause outcomes^17^^,^^18^ and to improve prognostic information provided by biomarkers.^19^ More recently, attention has moved to change in HRQoL using serial measures, which has shown similar associations in trial populations.^20^, ^21^, ^22^ To realise the potential of using HRQoL to assess prognosis, evidence is needed to guide clinical interpretation of HRQoL in different HF patients groups in routine clinical practice, but here the evidence is limited. The objective of this study was to examine the consistency of serial HRQoL measures and change as predictors of outcomes (all-cause mortality, all-cause hospitalization and HF hospitalization) according to sex, age and HF subtypes (HF with reduced, mildly reduced and preserved ejection fraction) in a prospective population based HF registry.

## Methods

### Population

The Swedish Heart Failure Registry (SwedeHF) is a national register enrolling patients with incident or prevalent HF admitted to hospitals (including private hospitals), attending outpatient clinics or primary care centres, in participating units. The registry has good coverage, with 84% of all hospitals entering data into the register.^23^ The register collects HRQoL data measured using the EuroQoL 5-dimensional questionnaire (EQ-5D) at baseline registrations and during subsequent visits, as well as patient demographics, clinical and healthcare information. Routine recording of EQ-5D at first registration was initiated in SwedeHF on February 1st 2008. We included all patients who had EQ-5D recorded at first registration who entered into the register on or after February 1st 2008 to 30th June 2019, aged 18 years or over. We excluded patients who had spurious death date information (n = 2) or who had changed or re-used personal identification numbers (n = 29). All patients were followed up for three years until the outcome event, migration out of Sweden, death or December 31st 2019, whichever came first.

### Ethics

Individual patient consent is not required for entry into the SwedeHF registry but patients are informed and can opt out. The registry was linked to the Cause of Death Registry and the National Patient Registry, providing the outcome data. Establishment of the SwedeHF registry and analysis of data was approved by the Swedish Ethical Review Authority (Ref: 2019-02698) and conforms to the Declaration of Helsinki.

### Health related quality of life measures

HRQoL was based on the Euro-QoL five dimensional visual analogue scale (EQ-5D-vas) which is a numerical scale between 0 (worst HRQoL imaginable) and 100 (best HRQoL imaginable).^24^ The EQ-5D is valid, comparable to other quality of life tools^25^^,^^26^ and is widely used in HF clinical trials.^27^ Based on the cut-offs for similar 0–100 HRQoL scales in prior studies,^28^^,^^29^ the EQ5D-vas scores are classified into four baseline categories: “best” (76–100), “good” (51–75), “bad” (26–50), and “worst” (0–25). In the registry, EQ5D-vas is measured at baseline and during a planned follow-up at approximately 1 year after baseline. EQ5D-vas is also repeated during subsequent registrations. For registrants with multiple EQ5D-vas measures after their baseline registration, we selected the closest measure to 1 year (median days 352; IQR 339, 378). Recognising that a change of more than 5 points is clinically meaningful,^9^^,^^29^ change in EQ5D-vas were grouped into five categories: “no significant change” (<5 points increase or decrease), “some improvement (5–9 points increase)” or “considerable improvement” (≥10 points increase); “some worsening” (5–9 points decrease)” or “considerable worsening” (≥10 points decrease).

Data was also collected on symptoms, including shortness of breath, fatigue, pain or discomfort, anxiety or depression, problems walking around, problems with activities of daily living and problems with self-care which were each coded as ‘any’ or ‘none’.

### Covariates

Age was categorised into 5-year categories as follows: ≤60, 61–65, 66–70, 71–75, 76–80, 81–85, >85 years. Left ventricular ejection fraction was registered as a categorical variable defined as reduced (HFrEF; <40%), mildly-reduced (HFmrEF; 40–49%) or preserved (HFpEF; ≥50%) (30).

Data was extracted on HF duration, previous HF hospitalization in one year, New York Heart Association (NYHA) class, NT-proBNP, systolic blood pressure, heart rate, estimated glomerular filtration rate (eGFR), hemoglobin, body mass index, potassium and drug therapies: beta blocker, renin–angiotensin system inhibitors (RASI; angiotensin converting enzyme inhibitor, angiotensin receptor blockers, angiotensin-receptor-neprilysin-inhibitor), mineralocorticoid receptor antagonists (MRA), digoxin, nitrates, diuretics, anti-platelets, anti-coagulants; interventions (revascularisation, devices;, lifestyle factors (smoking status); and comorbidities: myocardial infarction, dilated cardiomyopathy, valve disease, valve surgery, atrial arrhythmias, hypertension, diabetes, lung disease (COPD, asthma, tumours, fibrosis, tuberculosis or sarcoid).

### Outcomes

Hospitalization for any cause, hospitalization for HF and death from any cause. For HF admission we used the primary discharge code (ICD-10 I50.0, I50.1, I50.9, I09.9, I11.0, I13.0, I13.2).

### Statistics

Baseline covariates are presented by baseline EQ5D-vas categories as number (%) for categorical data, mean (standard deviation) for normally distributed continuous data and median (IQR) for skewed continuous data. Baseline EQ5D-vas and symptom measures are also presented by sex, age and HF subtype. The proportion of missing data was low for most covariates (<5%), but with a higher proportion for NT-proBNP, BMI and smoking (up to 33%) (Supplementary Table S1). To account for missing data in the covariates in the multivariable models, multiple imputations using chained equations were performed using MI Impute in Stata for all variables with missing data and results were obtained using Rubin's rules to combine 20 imputed datasets (31) MI imputation models included all covariates and outcomes used in the analyses (see Supplementary File for further details).

Using time since entry to the register as the timescale, we estimated time to hospitalization for any cause, hospitalization for HF and death from any cause using Royston-Parmar survival models (which estimate the baseline hazard function directly, using restricted cubic splines) (32) We compared baseline EQ5D-vas categories with the reference category (best HRQoL; 75–100). The change analysis was conducted in patients with a second EQ5D-vas measure recorded at approximately 1 year, using the time from the second measure as the starting time: all EQ5D-vas change measures were compared to the ‘no change’ reference group. For both analyses, unadjusted and adjusted hazard ratios with 95% confidence intervals, (CI) were estimated. For the adjusted analyses, all baseline covariates were added to the model. We tested for linearity in the continuous covariates by comparing different transformations (quadratic, log, splines) using likelihood ratio tests and used the transformation with the best model fit. Age was added as a time dependent covariate. We plotted age standardised cumulative incidence curves for each EQ5D-vas exposure measure and admission type, taking into account the competing risk of death, using the ‘standsurv’ command in Stata. We also plotted fitted probability and survival curves alongside non-parametrically estimated Kaplan Meier curves to observe model fit. To test the consistency of associations between the EQ5D-vas exposures and outcomes among different population groups, we entered a first order interaction term between the group (age, sex, HF subtype) and EQ5D-vas exposure into the models. To investigate whether any of the group interactions were significant, we used a joint test of the interaction coefficients using ‘mi test’ package in Stata-MP 16. Where the joint test was significant, (p-value <0.05), we performed stratified analyses for sub-categories of that group.

We tested the proportional hazards of the EQ5D-vas exposures by using Likelihood ratio tests to compare the adjusted models with and without the exposures included as a time-dependent covariate. Where there was evidence of an interaction with time for the exposures (p < 0.05), we reported effect estimates for each month of follow-up and plotted the estimated HR as a function of time. Finally, we performed two sensitivity analyses; (i) in patients without a prior HF admission in the previous year and (ii) using complete cases.

All analyses were conducted using Stata-MP 16. The level of statistical significance was set to 0.05, two-sided.

### Role of funding source

CL, Advanced Research Fellow, [NIHR-300111] is funded by the NIHR for this research project. This is independent research funded by NIHR and carried out at the National Institute for Health and Care Research (NIHR) Leicester Biomedical Research Centre (BRC). The views expressed are those of the author(s) and not necessarily those of the NIHR, NHS or the Department of Health and Social Care.

## Results

### Overall characteristics

Of the 79,917 registrants during the study timeframe, 23,533 patients had a baseline EQ5D-vas recorded and were included in the study; the median follow up time was 2.8 years (IQR 1.3–3), median age 74 (66–81) years, 8000 (34%) female, 3691 (17%) HFpEF, 5210 (24%) HFmrEF and 13,030 (59%) HFrEF (Table 1). Compared to registrants with a baseline EQ5D-vas recorded, those without, were more likely to be older (78 years; 69–85), female (41%), with HFpEF (27%) and in NYHA class III/IV (41%), with a higher symptom burden (Supplementary Table S2). There were 10,603 registrants with a follow-up EQ5D-vas recorded at a median of 352 days (IQR 339–378), who had similar characteristics to the overall group (Supplementary Table S3).

**Table 1:** Baseline characteristics by categories of Euro-Qol-5D Visual Analogue Scale (EQ5D-vas).

	All (n = 23,533)	Euro-Qol Visual Analogue Scale (EQ5D-vas)				p-value
		Best: 76–100 (n = 6794)	51–75 (n = 9751)	26–50 (n = 5971)	Worst: 0–25 (n = 1017)	
Age, median (IQR)	74 (66, 81)	72 (64, 79)	74 (66, 81)	75.0 (67, 82)	74 (65, 82)	<0.0001
Female	8000 (34)	1997 (29)	3294 (34)	2310 (39)	399 (39)	<0.0001
HF <6 months	13,148 (57)	4045 (61)	5484 (58)	3116 (53)	503 (51)	<0.0001
HF ≥6 months	9851 (43)	2576 (39)	4047 (42)	2738 (47)	490 (49)	
HF admission in the preceding year	9610 (41)	2496 (37)	3846 (39)	2717 (46)	551 (54)	<0.0001
Etiology						<0.0001
DCM	1213 (6)	434 (7)	457 (6)	268 (5)	54 (6)	
Other/Unknown	7302 (36)	2171 (37)	2989 (36)	1839 (36)	303 (36)	
Hypertension	4172 (21)	1220 (21)	1745 (21)	1049 (21)	158 (19)	
Ischemic Heart disease	6221 (31)	1770 (30)	2600 (32)	1609 (32)	242 (29)	
Heart valve disease	1113 (6)	291 (5)	446 (5)	302 (6)	74 (9)	
HF sub-type						
HFpEF	3691 (17)	987 (15)	1485 (16)	1031 (19)	188 (20)	<0.0001
HFmrEF	5210 (24)	1605 (25)	2167 (24)	1241 (23)	197 (21)	
HFrEF	13,030 (59)	3786 (59)	5462 (60)	3223 (59)	559 (59)	
NYHA III/IV	8218 (37)	1147 (18)	3183 (35)	3214 (58)	674 (73)	<0.0001
NT-proBNP, median (IQR)	2030 (895, 4250)	1649 (696, 3430)	2073 (939, 4199)	2350 (1047, 5180)	2779 (1142, 6218)	<0.0001
Systolic BP, mean (SD)	127 (21)	129 (20)	128 (21)	126 (21)	123 (22)	<0.0001
Heart rate, median (IQR)	70 (62, 80)	70 (60, 80)	70 (62, 80)	72 (63, 82)	73 (64, 85)	<0.0001
eGFR, mean (SD)	65 (22)	69 (21)	65 (21)	63 (23)	62 (24)	<0.0001
HB (g/L), mean (SD)	135 (17)	137 (17)	135 (17)	133 (17)	132 (18)	<0.0001
BMI, median (IQR)	27 (24, 31)	27 (24, 30)	27 (24, 30)	26.8 (24, 31)	27.8 (24, 32)	0.0012
Potassium, mean (SD)	4.228 (0.426)	4.237 (0.408)	4.229 (0.422)	4.226 (0.445)	4.174 (0.458)	<0.0001
Beta blocker	21,086 (90)	6075 (89)	8746 (90)	5372 (90)	893 (88)	0.21
RASI	21,252 (90)	6243 (92)	8862 (91)	5302 (89)	845 (83)	<0.0001
MRA	8034 (34)	2158 (32)	3318 (34)	2176 (37)	382 (38)	<0.0001
Digoxin	2728 (12)	669 (10)	1142 (12)	774 (13)	143 (14)	<0.0001
Nitrates	2333 (10)	498 (7)	971 (10)	730 (12)	134 (13)	<0.0001
Diuretic	16,650 (71)	4212 (62)	6995 (72)	4622 (78)	821 (81)	<0.0001
Statin	11,980 (51)	3442 (51)	5049 (52)	3015 (51)	474 (47)	0.0005
Anti platelet	9099 (39)	2666 (39)	3785 (39)	2274 (38)	374 (37)	0.37
Anti coagulant	11,575 (49)	3170 (47)	4850 (50)	3042 (51)	513 (51)	<0.0001
Revascularisation	6562 (29)	1862 (28)	2749 (29)	1679 (29)	272 (28)	0.49
Device	3258 (14)	851 (13)	1368 (14)	896 (15)	143 (14)	0.0005
Smoking Never	6531 (41)	1945 (43)	2732 (42)	1593 (40)	261 (38)	<0.0001
Smoking Previous	7497 (48)	2149 (47)	3142 (48)	1885 (47)	321 (47)	
Smoking current	1754 (11)	454 (10)	678 (10)	521 (13)	101 (15)	
Myocardial infarction	6366 (33)	1756 (30)	2662 (33)	1686 (34)	262 (32)	0.0003
Dilated cardiomyopathy	2728 (12)	910 (14)	1094 (12)	606 (11)	118 (12)	<0.0001
Valve disease	3830 (17)	975 (15)	1596 (17)	1040 (18)	219 (22)	<0.0001
Valve surgery	1461 (6)	380 (6)	653 (7)	348 (6)	80 (8)	0.0019
Atrial arrhythmia	11,509 (49)	3045 (45)	4825 (50)	3086 (52)	553 (55)	<0.0001
Hypertension	12,905 (56)	3594 (54)	5405 (57)	3349 (58)	557 (57)	0.0001
Diabetes	5256 (22)	1287 (19)	2140 (22)	1555 (26)	274 (27)	<0.0001
Lung disease	3413 (15)	689 (10)	1396 (15)	1090 (19)	238 (24)	<0.0001
Shortness of breath	19,767 (87)	4934 (75)	8429 (89)	5468 (95)	936 (95)	<0.0001
Fatigue	19,388 (85)	4651 (71)	8272 (88)	5520 (95)	945 (96)	<0.0001
Pain or discomfort	11,756 (52)	2104 (32)	4895 (52)	4026 (70)	731 (75)	<0.0001
Anxiety or depression	10,034 (44)	1415 (21)	4090 (43)	3783 (65)	746 (77)	<0.0001
Problems walking around	10,170 (44)	1553 (23)	4060 (43)	3815 (66)	742 (76)	<0.0001
Problems with ADL	7623 (33)	842 (13)	2848 (30)	3210 (55)	723 (75)	<0.0001
Problems with self-care	2734 (12)	325 (5)	849 (9)	1202 (21)	358 (37)	<0.0001

All results reported as n (%) unless otherwise stated. The denominator for the % figures include all complete data (see Supplementary Table S1 for missing data frequency). HF, heart failure; HFpEF, heart failure with preserved ejection fraction; HFmrEF, heart failure with mildly reduced ejection fraction; HFrEF, heart failure with reduced ejection fraction; NYHA, New York Heart Association; NT-BNP, NT-proB-type Natriuretic Peptide; eGFR, estimated glomerular filtration rate; HB, hemoglobin; BMI, body mass index; RASI, renin-angiotensin system inhibitors (comprising Angiotensin-converting enzyme inhibitors, Angiotensin receptor blockers and angiotensin Receptor-Neprilysin Inhibitor); MRA, Mineralocorticoid Receptor Antagonists; ADL, activities of daily living.

Compared to patients with the best HRQoL (EQ5D-vas 76–100), those with the worst HRQoL (EQ5D-vas 0–25) were more likely to be older, female, with longer duration of HF, more likely to have NYHA class III/IV, diabetes and COPD and less likely to be prescribed RASI. With the exception of sex, differences were similar for those experiencing considerable worsening of EQ5D-vas compared to those with no change (Supplementary Table S3). Worse baseline HRQoL was also associated with higher prevalence of symptoms; shortness of breath, fatigue, pain, anxiety/depression and problems with walking around, activities of daily living and self-care (Table 1; all p < 0.0001).

### Baseline symptoms and HRQoL by groups

**Fig. 1:**
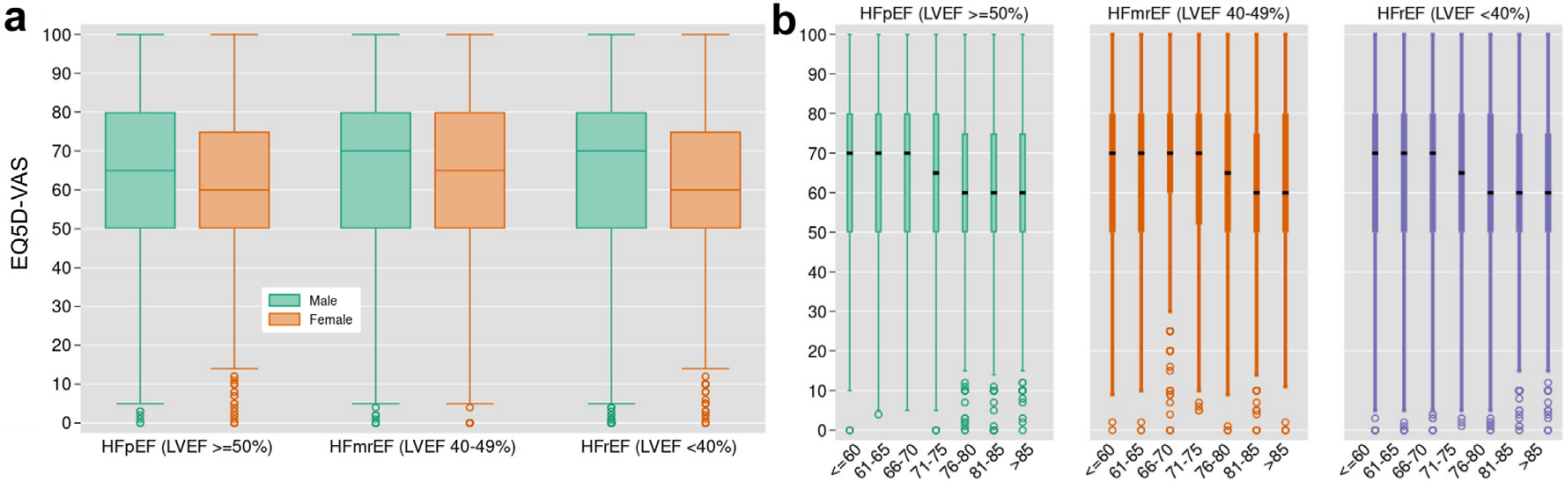
**Median baseline EQ5D-vas by HF subtype and (a) sex and (b) age**. Median EQ5D-vas by a) sex and b) age categories, for each HF subtype. The box plots show the interquartile range (25th–75th percentile), and median (horizontal line). The whiskers reach to 1.5 x interquartile range above and below the 25th and 75th percentile. The hollow circles are the outliers with extreme values of HRQoL that fall outside of the whisker range. EQ5D-vas, Euro-QoL visual analogue scale; HFpEF, heart failure with preserved ejection fraction; HFmrEF, heart failure with mildy-reduced ejection fraction; HFrEF, heart failure with reduced ejection fraction.

Median baseline EQ5D-vas was lower (worse) in females (60; IQR 50–30) than males (70; 50–80) (Table 2) and this was consistent across different HF subtypes (Fig. 1a). Median EQ5D-vas was also lower in the oldest (60; 50–75) than youngest group (70; 50–80) and in people with HFpEF (62; 50–80) than HFmrEF (70; 50–80) and HFrEF (65; 50–80) (Table 2). There was a gradual decrease in EQ5D-vas score for each increase in age category, which was consistent among people with different HF subtypes (Fig. 1b). The most prevalent symptoms at first registration were shortness of breath (19,767; 87%), fatigue (19,388; 85%) and pain (11,756; 52%). Symptom prevalence and problems with self-care, walking and activities of living, were significantly higher in the groups with the worst HRQoL (female, oldest and HFpEF), compared to those with better HRQoL (male, youngest and HFrEF) (Table 2). In terms of the 10,603 patients with a follow-up EQ5D-vas, 865 (8%) of patients experienced some improvement, 3944 (37%) experienced considerable improvement, 672 (6%) experienced some worsening and 3041 (29%) experienced considerable worsening (Supplementary Table S4). Results were similar by sex albeit more men (626/7128; 9%) than women (239/3475; 7%) experienced some improvement. Higher rates of considerable worsening was experienced by the oldest (323/835; 39%) compared to the youngest (365/1623; 22%) groups and in people with HFpEF (474/1427; 33%) compared to people with HFmrEF (680/2341; 29%) or HFrEF (1646/6128; 27%).

**Table 2:** Baseline EQ5D components and EQ5D-vas by groups.

Factor	Male (n = 15,533)	Female (n = 8000)	Youngest (≤60 years) (n = 3521)	Oldest (>85 years) (n = 2376)	HFpEF (n = 3691)	HFmrEF (n = 5210)	HFrEF (n = 13,030)
EQ5D-VAS, median (IQR)	70 (50, 80)	60 (50, 75)	70 (50, 80)	60 (50, 75)	62.0 (50, 80)	70 (50, 80)	65 (50, 80)
Shortness of breath	12,889 (86)	6878 (89)	2791 (82)	2064 (90)	3165 (89)	4301 (85)	10,989 (87)
Fatigue	12,519 (83)	6869 (89)	2687 (79)	2101 (91)	3126 (88)	4226 (83)	10,718 (85)
Pain or discomfort	7152 (47)	4604 (59)	1511 (44)	1376 (60)	2154 (60)	2680 (53)	6035 (48)
Anxiety or depression	5956 (40)	4078 (53)	1751 (51)	991 (43)	1657 (46)	2186 (43)	5524 (44)
Problems walking around	5995 (40)	4175 (54)	873 (25)	1556 (67)	2012 (56)	2202 (43)	5053 (40)
Problems performing ADL	4533 (30)	3090 (40)	992 (29)	1051 (46)	1353 (38)	1546 (30)	4107 (32)
Problems with self care	1580 (10)	1154 (15)	194 (6)	599 (26)	594 (17)	551 (11)	1284 (10)

All results reported as n (%) unless otherwise stated. HF, heart failure; HFpEF, heart failure with preserved ejection fraction; HFmrEF, heart failure with mildly reduced ejection fraction; HFrEF, heart failure with reduced ejection fraction; EQ5D-vas, Euro-QoL visual analogue scale; ADL, activities of daily living.

All group differences were significant (p < 0.0001).

### Associations with outcomes

During a total follow up of 51,418 person years, there were 15,004 first any-cause admissions, 5555 first HF admissions and 4697 deaths with incidence rates (IR) of 518, 126 and 91 per 1000 person years, respectively.

### Any admission by baseline HRQoL and change scores

**Fig. 2:**
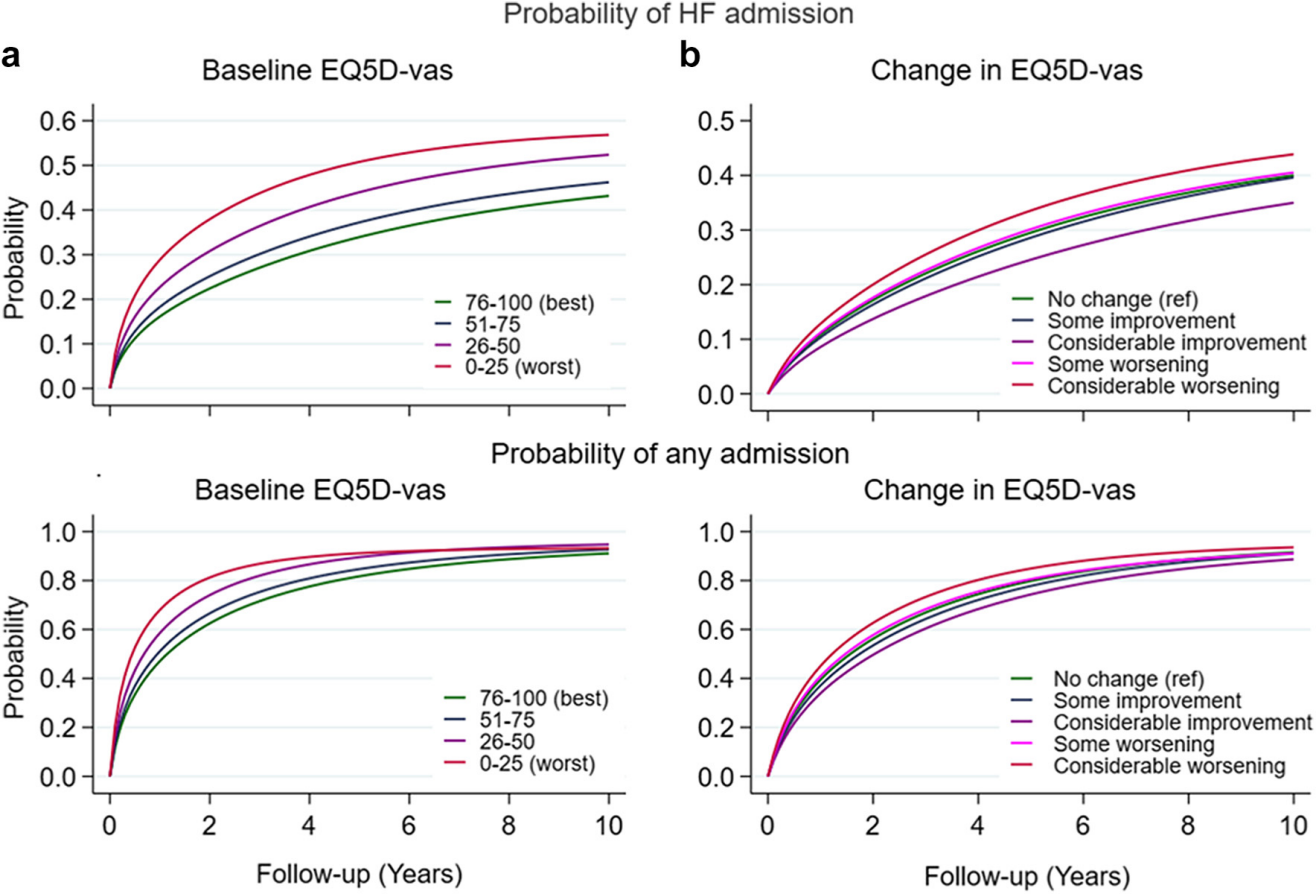
**Age standardised probability of admission, accounting for competing risk of death, during follow-up by (a) baseline EQ5D-vas and (b) change in EQ5D-vas**. Age standardised cumulative incidence curves, taking account of competing risk of death, for each category of EQ5D-vas exposure a) at baseline and b) from change after one year. EQ5D-vas, Euro-QoL visual analogue scale.

Lower baseline EQ5D-vas score was associated with a significantly higher rates of admission for any cause, independently of patient and clinical characteristics. In the fully adjusted models, compared to the highest EQ5D-vas score (76–100), a score of 26–50 was associated with 27% higher rates (HR 1.27; 1.21–1.33) and the lowest VAS score (0–25) was associated with 39% higher rates (HR 1.39; 1.28–1.51) (Table 3, Fig. 2a, Supplementary Figure S1). There was a significant interaction between baseline HRQoL and age (p = 0.005). In the stratified analyses, there appeared to be some attenuation of the associations between the lowest levels of HRQoL in the oldest age group (>85 years) (Supplementary Table S5). There were no interactions between baseline HRQoL and sex or HF sub-type. Compared to patients with no significant change in EQ5D-vas, those with a considerable improvement in EQ5D-vas score over a year (≥10 points), had a lower rate of admissions for any cause (HR 0.75; 0.70, 0.81) and those with a considerable worsening of EQ5D-vas score, had higher rates (HR 1.25; 1.16, 1.35), independently of baseline EQ5D-vas and other risk factors (Table 3, Fig. 2b Supplementary Figure S2). There were no significant interactions between change in EQ5D-vas and age, sex or HF subtype in the adjusted models (p > 0.05), albeit there was a pattern of a reduced association in women with considerable worsening of EQ5D-vas, compared to men (Supplementary Table S6).

**Table 3:** Outcomes by baseline EQ5D-vas and change.

EQ5D-vas at baseline (n = 23,533)	1st hospitalization for any cause			1st hospitalization for HF			Death		
	Incidence rate (per 1000 py)	Unadjusted	Adjusted	Incidence rate (per 1000 py)	Unadjusted	Adjusted	Incidence rate (per 1000 py)	Unadjusted	Adjusted
		HR (95 CI)			HR (95 CI)			HR (95 CI)	
76–100 (best)	387	1.0	1.0	89	1.0	1.0	59	1.0	1.0
51–75	507	1.28 (1.23, 1.33)	1.09 (1.04, 1.14)	117	1.30 (1.21, 1.40)	1.01 (0.94, 1.09)	86	1.47 (1.36, 1.60)	1.15 (1.06, 1.25)
26–50	697	1.67 (1.60, 1.75)	1.27 (1.21, 1.33)	178	1.91 (1.78, 2.06)	1.22 (1.12, 1.31)	129	2.21 (2.04, 2.40)	1.37 (1.26, 1.49)
0–25 (worst)	878	2.05 (1.89, 2.21)	1.39 (1.28, 1.51)	216	2.27 (2.01, 2.56)	1.22 (1.07, 1.38)	167	2.85 (2.51, 3.23)	1.50 (1.31, 1.72)

**EQ5D-vas change over 1 year (n = 10,603)**		**Unadjusted**	**Adjusted**^a^		**Unadjusted**	**Adjusted**^a^		**Unadjusted**	**Adjusted**^a^
		HR (95 CI)			HR (95 CI)			HR (95 CI)	
No change	386	1.00 (Ref)	1.00 (Ref)	87	1.00 (Ref)	1.00 (Ref)	79	1.00 (Ref)	1.00 (Ref)
Improve 5–9 pts	328	0.85 (0.77, 0.95)	0.91 (0.82, 1.01)	76	0.88 (0.73, 1.06)	0.95 (0.78, 1.15)	60	0.75 (0.61, 0.92)	0.84 (0.68, 1.03)
Improve >10 pts	328	0.69 (0.64, 0.74)	0.75 (0.70, 0.81)	70	0.62 (0.55, 0.71)	0.71 (0.62, 0.81)	65	0.60 (0.52, 0.68)	0.71 (0.62, 0.81)
Worse 5–9 pts	387	1.07 (0.95, 1.20)	1.04 (0.92, 1.16)	86	1.06 (0.87, 1.30)	0.93 (0.76, 1.14)	74	1.00 (0.82, 1.23)	0.94 (0.77, 1.16)
Worse >10 pts	512	1.38 (1.28, 1.48)	1.25 (1.16, 1.35)	120	1.47 (1.30, 1.67)	1.25 (1.10, 1.41)	118	1.63 (1.45, 1.84)	1.37 (1.20, 1.55)

HF, heart failure; EQ5D-vas, Euro-QoL visual analogue scale; pts, points.

All models adjusted for age, sex, HF type, HF duration, previous HF admission in the preceding year, etiology, NYHA, NTpro-BNP, systolic blood pressure (SBP), heart rate (HR), estimated glomerular filtration rate (eGFR), hemoglobin (HB), BMI, potassium, BB, RASI (comprising Angiotensin-converting enzyme inhibitors, Angiotensin receptor blockers and angiotensin Receptor-Neprilysin Inhibitor), MRA, digoxin, nitrates, diuretic, anti-coagulant, anti-platelet, revascularisation, device, smoking, myocardial infarction (MI), dilated cardiomyopathy, valve disease, valve surgery, atrial arrhythmia, hypertension, diabetes, lung disease.

a EQ5D-VAS change models also adjusted for baseline EQ5D-VAS.

### HF admission by baseline HRQoL and change scores

Results were similar for rates of admission for HF. Compared to the highest EQ5D-vas score (76–100), a score of 26–50 was associated with 22% higher rates (HR 1.22; 1.12, 1.31). Associations were similar for the lowest EQ5D-vas score (0–25) (HR 1.22; 1.07, 1.38) (Table 3, Fig. 2a, Supplementary Figure S1). There were no interactions between baseline HRQoL and age, sex or HF subtype, albeit the pattern of diminished associations between HRQoL and HF admission in the oldest age group, remained (Supplementary Table S5). Compared to patients with no significant change in EQ5D-vas, patients with a considerable improvement in EQ5D-vas score over a year (>10 points increase) had 29% lower rates of HF admission (HR 0.71; 0.62, 0.81) while those with a considerable worsening had 25% higher rates (HR 1.25; 1.10, 1.41), independently of baseline EQ5D-vas and other risk factors (Fig. 2b, Supplementary Figure S2). There was some evidence of an interaction between change in HRQoL and sex (p = 0.033). In the stratified analyses, the association between considerable worsening and HF admission was attenuated and non-significant in women; HR 1.10 (0.88, 1.39), but strengthened in men; HR 1.30 (1.11, 1.51) (Supplementary Table S6).

### Death by baseline HRQoL and change scores

**Fig. 3:**
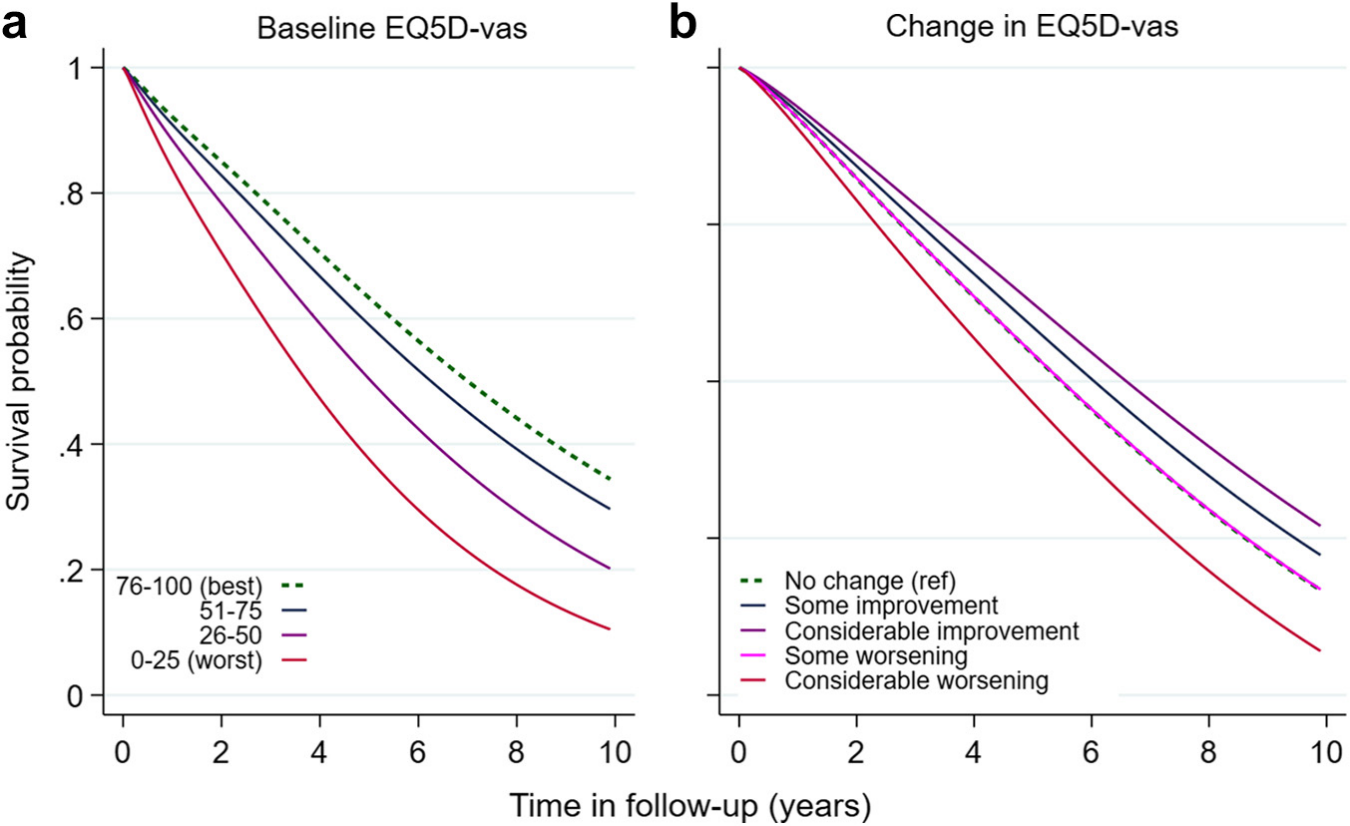
A**ge standardised survival by (a) baseline EQ5D-vas and (b) change in EQ5D-vas**. Age-standardised survival curves, for each category of EQ5D-VAS exposure a) at baseline and b) from change after one year. For change in EQ5D-VAS, curves were also standardised at mean baseline VAS. EQ5D-vas, Euro-QoL visual analogue scale.

Compared to the group with best EQ5D-vas, there were incrementally higher rates of death, with each worsening EQ5D-vas category (Fig. 3a), reaching HR 1.50 (1.31, 1.72) in the worst EQ5D-vas (0–25) group (Table 3). Change in EQ5D-vas also remained a consistent predictor, with considerable improvement in EQ5D-vas associated with lower rates of death (HR 0.71; 0.62, 0.81) and considerable worsening in EQ5D-vas with higher rates (HR 1.37; 1.20, 1.55) (Fig. 3b, Supplementary Figure S3). There were no interactions with sex, age or HF subtype.

### Time dependent associations

There was no evidence of a time dependent association between most of the categories of baseline EQ5D-vas or change in EQ5D-vas and outcomes (p > 0.05). However, there was evidence of time-dependency for the most severe categories of both baseline EQ5D-vas (0–25) and change in EQ5D-vas (>10 points worsening). Estimates were higher in the first few months of follow-up and gradually decreased over time. Between month 1 and month 36 of follow-up, estimates for worst baseline EQ5D-vas, ranged from HR 1.63 (1.47, 1.80) to 1.06 (0.93, 1.24) for hospitalization for any cause, 1.44 (1.22, 1.70) to 0.94 (0.76, 1.16) for hospitalization for HF and 3.36 (2.58–4.63) to 0.98 (0.80, 1.19) for death (see Supplementary Table S7 for estimates and HR plots). Over the same follow-up time estimates for considerable worsening in EQ5D-vas, ranged from HR 1.38 (1.23, 1.56) to 1.16 (1.03, 1.30) for any-cause admission and 1.63 (1.28, 2.07) to 1.06 (0.87, 1.29) for HF admission. There was no time dependent effect between considerable worsening of ED5D-vas and death (see Supplementary Table S8 for estimates and HR plots).

### Sensitivity analyses

In the first sensitivity analysis, removing patients with a HF prior admission in one year, the estimates of association were similar and slightly strengthened between baseline EQ5D-vas and outcomes (Supplementary Table S9).

In the second sensitivity analysis using complete cases, estimates showed similar and often stronger associations between EQ5D-vas and outcomes, but with less precision (Supplementary Table S10).

## Discussion

Compared to echocardiography and invasive biomarkers, HRQoL is a pragmatic, patient-centred and potentially low-cost indicator of clinical status and prognosis. Following recent calls to integrate serial recordings of HRQoL into routine care, our study adds new evidence on the relationship between changing HRQoL and outcomes among a heterogeneous population with HF. In summary, we found that women, people who were older or who had HFpEF reported significantly worse HRQoL and higher symptom burden than their respective counterparts. However, despite these differences, with the exception of worsening HRQoL in women for HF admission, HRQoL and its change over time were consistent and independent predictors of worse outcomes in all groups. Furthermore, risk in those with the lowest HRQoL and in those with considerable worsening of HRQoL over time, was highest in the first 12-months following measurement. These novel findings could assist clinicians in their interpretation of patient reported HRQoL during clinical consultation and to facilitate risk assessment and care planning.

Most prior evidence has focused on the relationship between baseline HRQoL status and outcomes in HF using small HFrEF (16–18, 33, 34) and trial populations (12, 35) as well as composite outcomes. All have reported a significant association between worse HRQoL and increased risk. More recently, the G-CHF (Global Congestive Heart Failure) general population study presented results on baseline HRQoL similar to our own findings, (36) showing a dose–response relationship between worse categories of HRQoL and admission for HF and risk of death in people with HFpEF and HFrEF. Few studies have investigated change in HRQoL status and outcomes in HF; moreover, all prior evidence is limited to HFrEF trials, with EPHESUS (37), ESCAPE (38) and HF-ACTION (22) all reporting an independent association between change in HRQoL and the composite of HF admission or death. We found that a 10-point reduction in EQ5D-vas (worsening HRQoL) was consistently associated with increased rates of any-cause admission and death in the general HF population, irrespective of age, sex and HF-subtype. Conversely, improving HRQoL was associated with reduced rates. Our findings also add to prior reports by showing a more complex relationship between HRQoL and outcomes. Both the lowest baseline HRQoL and considerable worsening of HRQoL were associated with the highest risk of outcomes in the first year of follow-up time, showing the sensitivity of HRQoL for identifying patients at imminent risk. This is important information for identifying the highest risk patients for early intervention.

Whilst variation in HRQoL among population groups with HF has been well reported, much less is the evidence on how these differences influence its associations with outcomes. In our study, baseline HRQoL was significantly worse in women than men, in line with previous reports (39–45) HRQoL was also worse in patients with HFpEF than with HFrEF but, in this respect, prior reports are conflicting. In the CHARM (46) and COACH (47) trials HRQoL was similar in both HF groups, whilst the G-CHF study found those with HFpEF had better HRQoL. All three studies categorised HFpEF as an ejection fraction of >40%. The HFmrEF group in our study (EF 40–49%) had the best HRQoL in contrast to the HFpEF group (EF ≥50%) with worst HRQoL. Due to the large sample size, we were also able to categorise age by 5-year intervals. Previous studies have shown worse HRQoL in younger people (often associated with higher levels of anxiety and depression) (45, 48). These studies have included younger populations with an oldest category of around age 70 years or more. We found that although HRQoL improved from the youngest age category to the age 66–70 years category, it worsened thereafter, alongside increasing, prevalence of anxiety and depression. These findings present new evidence on worse HRQoL in HFpEF and in the oldest age groups, which may have been previously masked, by less sensitive categorisation.

Despite these differences in baseline HRQoL, this is the first study to our knowledge to show that worse HRQoL is a consistent and independent predictor of increased risk in all age, sex and HF subtype groups and for several outcomes. However, there were some differences in the strength of associations between groups. Whilst baseline HRQoL was worse in the older compared to the younger group, the associations between bad and ‘worst’ HRQoL and admissions were diminished in the older age groups. Likewise, whilst women had worse baseline HRQoL compared to men, worsening HRQoL in women was not associated with HF admission and had a reduced association with all-cause admission, compared to men. Part explanation for these findings may be the lower HRQoL scores in women and oldest age group at baseline. Lower HRQoL could create a ‘floor effect’, whereby a tool may be less able to discriminate between meaningful changes in health states. However, there has also been prior reports of women devaluing their symptoms (49, 50,) Higher symptom resilience may influence reporting of HRQoL, resulting in less differentiation among women and diminished association with outcomes. These findings suggest the importance of including both baseline HRQoL and its change in risk assessments, particularly for the sickest patients, where change may be less sensitive.

Collectively, these findings indicate that HRQoL and its change over time are important prognostic indicators, among people with HF from different age and sex groups and in people with HFrEF, HFmrEF and HFpEF. Unlike other clinical status measures, change in HRQoL is standardised to the patient and this less susceptible to measurement error. That said, routine measurement of HRQoL is not currently embedded in clinical care or patient self–care pathways. Symptom and health monitoring by patients with HF is often ad hoc and compounded by comorbidities and low health literacy (51) Lack of time and suitable information technology also create barriers to implementation in clinical practice (52) Recently there has been a move to using single-item ‘global’ questions to assess HRQoL to substantially reduce patient and healthcare resource burden (53) We used a single-item HRQoL measure, which consisted of a 100 point visual analogue scale. This single-item score is simple and quick for patients to complete, making it accessible among patients from different social backgrounds. Importantly, given the exponential increase in admissions for non-cardiovascular causes in patients with HF over recent years, (54) the EQ5D-vas was associated with both HF and all-cause outcomes. Essentially, we also show that serial measures of HRQoL can provide important and additional prognosis information. In HF, worsening of HRQoL could act as a valuable trigger for optimising treatments or referral to specialist services.

Our findings are from a routine real-world register of people with HF from most hospitals in Sweden, as well as from primary care and covering the range of HF patients often excluded from clinical trials. Clinical diagnoses and outcomes are well validated and HRQoL is measured at baseline and during follow-up. The large sample meant that we could closely and robustly examine the relationship between HRQoL and a range of outcomes in detailed age, sex and HF subtype groups, also accounting for competing risk of death. Given the routine collection of data, there was some missingness across the variables, which led to high combined missingness in the multivariable models. However, we used multiple imputation and results were consistent with the complete case analysis. Not all patients had HRQoL status recorded, so we cannot rule out selection or measurement bias or account for unmeasured or unknown confounding. HRQoL was recorded at baseline and 1-year follow-up, and covariates were recorded at baseline, so we were unable to account for variations in HRQoL or covariates, during different time-periods. Further work is needed to understand more longitudinal variations in HRQoL and ethno-economic variations.

In patients with HF, HRQoL and its change over time are independent indicators of admission and death in HF patients irrespective of age, sex and ejection fraction. Serial recordings of HRQoL in routine care have the potential to provide a simple, cost effective and patient-centred approach to prognosis and care planning.

## Contributors

All authors read and approved the final version of the manuscript. CL had full access to all the data in the study and takes responsibility for the integrity of the data, the accuracy of the data analysis and decision to submit the manuscript. LB also had direct access to the underlying data reported in the manuscript. CL: Funding acquisition, Conceptualization, Data curation, Formal Analysis, Methodology, Writing—original draft; LB: Data curation & validation, supervision, writing—review & editing; AS: Conceptualization, supervision, writing—review & editing; UD/LL/GS: conceptualization, data curation, project administration, resources, writing—review & editing; IS/FZ/MA/SH/UK/WT/CSPL/KK: conceptualization, writing—review & editing.

## Data sharing statement

Access to the data is by request to the Head of SwedeHF research foundation, professor Ulf Dahlström (ulf.dahlstrom @ rikssvikt.se).

## Declaration of interests

LHL is supported by Karolinska Institutet, the Swedish Research Council [grant 523-2014-2336], the Swedish Heart Lung Foundation [grants 20150557, 20190310], and the Stockholm County Council [grants 20170112, 20190525], and reports research grants, consulting fees and payment or honoraria from Merck, Vifor Pharma, AstraZeneca, Bayer, Pharmacosmos, MedScape, Sanofi, Lexicon, Myokardia, Boehringer Ingelheim, Servier, Novartis, Boston Scientific, Abbott, MedScape, Radcliffe and stock ownership in AnaCardio. CSPL is supported by a Clinician Scientist Award from the National Medical Research Council of Singapore and reports research grants, consulting fees and payment or honoraria from National Medical Research Council of Singapore, NovoNordisk, Roche Diagnostics, Alleviant Medical, Allysta Pharma, Amgen, AnaCardio AB, Applied Therapeutics, AstraZeneca, Bayer, Boehringer Ingelheim, Boston Scientific, Bristol Myers Squibb, CardioRenal, Cytokinetics, Darma Inc., EchoNous Inc, Eli Lilly, Impulse Dynamics, Intellia Therapeutics, Ionis Pharmaceutical, Janssen Research & Development LLC, Medscape/WebMD Global LLC, Merck, Novartis, Novo Nordisk, Prosciento Inc, Quidel Corporation, Radcliffe Group Ltd., Recardio Inc, ReCor Medical, Roche Diagnostics, Sanofi, Siemens Healthcare Diagnostics and Us2.ai and serves as co-founder & non-executive director of Us2.ai. UD reports research grants, consulting fees and payment or honoraria from Astra Zeneca, Vifor, Pfizer, Boehringer-Ingelheim, Boston Scientific, Roche Diagnostics and Amgen. All outside the submitted manuscript.

GS reports research grants, consulting fees and payment or honoraria from TEVA, Medical Education Global Solutions, Genesis, Agence Recherche (ANR), Vifor Pharma, Boehringer Ingelheim, AstraZeneca, Merck, Cytokinetics, Servier, Medtronic, Dynamicom Education, INTA, Uppsala Clinical Research Center (UCR), Boehringer Ingelheim.

KK reports research grants, consulting fees and payment or honoraria from Applied Therapeutics and Roche.

The remaining authors have nothing to disclose.
