# Supplementary material for: Changing health related quality of life and outcomes in heart failure by age, sex and subtype
Source: eClinicalMedicine. 2023 Sep 14;64:102217. doi: 10.1016/j.eclinm.2023.102217 (PMC10514432; doi:10.1016/j.eclinm.2023.102217)
Supplement: Supplementary File [file mmc1.docx]

**SUPPLEMENTARY MATERIAL**

**Changing health-related quality of life and outcomes in heart failure by age, sex and subtype.**

Lawson CA, Benson L, Squire I, Zaccardi F, Ali M, Hand S, Kadam U, Tay WT, Dahlstrom U, Lund LH, Savarese G, Lam CSP, Khunti K, Strömberg A

**Table of Contents**

[S1 Table: Missing data 2](#_Toc139807215)

[Imputation methods 3](#_Toc139807216)

[S2 Table: Baseline characteristics by recording of Euro-Qol-5D Visual Analogue Scale (EQ5D-vas) 4](#_Toc139807217)

[S3 Table: Baseline characteristics by categories of change in Euro-Qol-5D Visual Analogue Scale (EQ5D-vas) 6](#_Toc139807218)

[S4 Table: Change in Euro-Qol Visual Analogue Scale (EQ5D-vas) by population groups (sex, age and HF sub-type) 8](#_Toc139807219)

[S5 Table: Interaction between EQ5D-vas and age 9](#_Toc139807220)

[S6 Table: Interaction between EQ5D-vas change and sex 10](#_Toc139807221)

[S7 Table: Time dependent association between lowest EQ5D-vas (0-25) and outcomes 11](#_Toc139807222)

[S8 Table: Time dependent association between considerable worsening of EQ5D-vas (>10 points) and outcomes 13](#_Toc139807223)

[S9 Table: Sensitivity analysis, removing patients with an admission prior to the change analysis. 15](#_Toc139807224)

[S10 Table: Sensitivity analysis using complete cases. 16](#_Toc139807225)

[S1 Figure: Probability of admission by baseline EQ5D-vas (fitted versus non-parametric Kaplan Meier curves) 17](#_Toc139807226)

[S2 Figure: Probability of admission by EQ5D-vas change (fitted versus non-parametric Kaplan Meier curves) 18](#_Toc139807228)

[S3 Figure: Survival curves for EQ5D-vas exposures (fitted versus non-parametric Kaplan Meier curves) 19](#_Toc139807230)

[Supplementary references (30-56): 20](#_Toc139807232)

| S1 Table: Missing data | |  |
| --- | --- | --- |
|  | Missing n (%) |  |
| Age | - |  |
| Sex | - |  |
| HF duration | 534 (2.3) |  |
| HF admission in previous year | - |  |
| Etiology | 3512 (14.9) |  |
| LVEF | 1602 (6.8) |  |
| NYHA III/IV | 1470 (6.2) |  |
| NT-proBNP | 7060 (30.0) |  |
| Systolic BP | 96 (0.4) |  |
| Heart rate | 262 (1.1) |  |
| eGFR | 315 (1.3) |  |
| HB (g/L), | 1230 (5.2) |  |
| BMI | 6280 (26.7) |  |
| Potassium | 1930 (8.2) |  |
| Beta blocker | 38 (0.2) |  |
| RASI | 18 (0.1) |  |
| MRA | 96 (0.4) |  |
| Digoxin | 56 (0.2) |  |
| Nitrates | 86 (0.4) |  |
| Statin | 53 (0.2) |  |
| Diuretic | 69 (0.3) |  |
| Anti-platelet | 89 (0.4) |  |
| Anti-coagulant | 63 (0.3) |  |
| Revascularisation | 680 (2.9) |  |
| Device | 463 (2.0) |  |
| Smoking | 7751 (32.9) |  |
| Myocardial infarction | 3974 (16.9) |  |
| Dilated cardiomyopathy | 1065 (4.5) |  |
| Valve disease | 695 (3.0) |  |
| Valve surgery | 551 (2.3) |  |
| Atrial arrhythmia | 132 (0.6) |  |
| Hypertension | 528 (2.2) |  |
| Diabetes | 92 (0.4) |  |
| COPD | 374 (1.6) |  |
| Shortness of breath | 747 (3.2) |  |
| Fatigue | 768 (3.3) |  |
| Pain or discomfort | 717 (3.0) |  |
| Anxiety or depression | 731 (3.1) |  |
| Problems walking around | 616 (2.6) |  |
| Problems with ADL | 694 (2.9) |  |
| Problems with self-care | 641 (2.7) |  |
| HF, heart failure; LVEF, left ventricular ejection fraction; NYHA, New York Heart Association; NT-proBNP, NT-proB-type Natriuretic Peptide; eGFR, estimated glomerular filtration rate; HB, hemoglobin; BMI, body mass index; RASI, renin-angiotensin system inhibitors (comprising Angiotensin-converting enzyme inhibitors, Angiotensin receptor blockers and angiotensin Receptor-Neprilysin Inhibitor); MRA, Mineralocorticoid Receptor Antagonists; COPD, chronic obstructive pulmonary disease; ADL, activities of daily living | | |

# **Imputation methods**

For the imputation models we used the following approach:

Where there was evidence of non-linearity (BP, age, eGFR and BMI) or interactions (age##VAS; sex##VAS_change), we used the transform and then impute method recommended by von Hippel (<https://journals.sagepub.com/doi/10.1111/j.1467-9531.2009.01215.x>) in the imputation model.

For the time dependant outcomes, we added the primary outcome indicator (e.g all-cause hospitalisation) and the predicted cumulative hazard from the model (White IR, Royston P. Imputing missing covariate values for the Cox model. Stat Med. 2009 Jul 10;28(15):1982-98.).

Logistic (binary, ordered or polytomous) or predictive mean matching was used to predict the missing categorical or continuous variables respectively. Predictive mean matching estimates the missing values by matching observations to their closest observed values and reduces the risk of predictions outside of the observed range. Imputation was performed for the missing covariate data and not for the main exposures or outcomes.

Given that the change analysis was conducted in a sub-set of survivors at 1 year, we imputed the data for the baseline and change analyses separately.

| S2 Table: Baseline characteristics by recording of Euro-Qol-5D Visual Analogue Scale (EQ5D-vas) | | |
| --- | --- | --- |
|  |  |  |
|  | EQ5D-vas at baseline | |
|  | No  N=56,384 | Yes  N=23,533 |
| Age, median (IQR) | 78 (69, 85) | 74 (66, 81) |
| Female | 23139 (41%) | 8000 (34%) |
| HF <6 months | 27265 (50%) | 13148 (57%) |
| HF >= 6 months | 26765 (50%) | 9851 (43%) |
| Etiology |  |  |
| DCM | 1865 (4%) | 1213 (6%) |
| Other/Unknown | 16924 (38%) | 7302 (36%) |
| Hypertension | 9474 (22%) | 4172 (21%) |
| Ischemic Heart disease | 12842 (29%) | 6221 (31%) |
| Heart valve disease | 2861 (7%) | 1113 (6%) |
| HF subtype |  |  |
| HFpEF | 12999 (27%) | 3691 (17%) |
| HFmrEF | 10869 (23%) | 5210 (24%) |
| HFrEF | 23702 (50%) | 13030 (59%) |
| NYHA III/IV | 12242 (41%) | 8218 (37%) |
| NT-proBNP, median (IQR) | 2723 (1157, 6200) | 2030 (895, 4250) |
| Systolic BP, mean (SD) | 129 (22) | 127 (21) |
| Heart rate, median (IQR) | 74 (64, 86) | 70.0 (62, 80) |
| eGFR, mean (SD) | 61 (24) | 65 (22) |
| HB (g/L), mean (SD) | 130 (18) | 135 (17) |
| BMI, median (IQR) | 26 (23, 30) | 27 (24, 31) |
| Potassium, mean (SD) | 4.2 (0.5) | 4.2 (0.4) |
| Beta blocker | 47459 (85%) | 21086 (90%) |
| RASI | 43976 (78%) | 21252 (90%) |
| MRA | 16658 (30%) | 8034 (34%) |
| Digoxin | 7362 (13%) | 2728 (12%) |
| Nitrates | 7456 (13%) | 2333 (10%) |
| Diuretic | 42969 (77%) | 16650 (71%) |
| Statin | 24908 (44%) | 11980 (51%) |
| Anti Platelet | 32225 (58%) | 14345 (61%) |
| Anti coagulant | 31922 (57%) | 11895 (51%) |
| Revascularisation | 40531 (74%) | 16291 (71%) |
| Device | 47321 (86%) | 19812 (86%) |
| Smoking Never | 7775 (14%) | 3258 (14%) |
| Smoking Previous | 11798 (46%) | 6531 (41%) |
| Smoking current | 10584 (41%) | 7497 (48%) |
| Myocardial infarction | 28595 (67%) | 13193 (67%) |
| Dilated cardiomyopathy | 48918 (91%) | 19740 (88%) |
| Valve disease | 42966 (79%) | 19008 (83%) |
| Valve surgery | 51572 (94%) | 21521 (94%) |
| Atrial arrhythmia | 26561 (47%) | 11892 (51%) |
| Hypertension | 23095 (42%) | 10100 (44%) |
| Diabetes | 41699 (75%) | 18185 (78%) |
| COPD | 45118 (82%) | 19746 (85%) |
| Shortness of breath | 1160 (16%) | 3019 (13%) |
| Fatigue | 1195 (17%) | 3377 (15%) |
| Pain or discomfort | 3602 (57%) | 11060 (48%) |
| Anxiety or depression | 3491 (61%) | 12768 (56%) |
| Problems walking around | 3980 (55%) | 12747 (56%) |
| Problems with ADL | 4742 (67%) | 15216 (67%) |
| Problems with self care | 5847 (82%) | 20158 (88%) |
| All results reported as n (%) unless otherwise stated. HF, heart failure; HFpEF, heart failure with preserved ejection fraction; HFmrEF, heart failure with mildy-reduced ejection fraction; HFrEF, heart failure with ; reduced ejection fraction; NYHA, New York Heart Association; NT-BNP, NT-proB-type Natriuretic Peptide; eGFR, estimated glomerular filtration rate; HB, hemoglobin; BMI, body mass index; RASI, renin-angiotensin system inhibitors (comprising Angiotensin-converting enzyme inhibitors, Angiotensin receptor blockers and angiotensin Receptor-Neprilysin Inhibitor); MRA, Mineralocorticoid Receptor Antagonists; COPD, chronic obstructive pulmonary disease; ADL, activities of daily living | | |

| S3 Table: Baseline characteristics by categories of change in Euro-Qol-5D Visual Analogue Scale (EQ5D-vas) | | | | | | | |
| --- | --- | --- | --- | --- | --- | --- | --- |
|  | **Change in Euro-Qol Visual Analogue Scale (EQ5D-VAS)** | | | | | |  |
|  | All (n=10,603) | No change (n=2,081) | Some improvement (n=865) | Considerable improvement (n=3,944) | Some worsening: (n=672) | Considerable worsening: (n=3,041) | p-value |
| Age, median (IQR) | 73 (65, 80) | 73 (65, 80) | 72 (64, 79) | 72 (64, 79) | 73 (66, 80) | 74 (67, 81) | <0.0001 |
| Female | 3475 (33%) | 684 (33%) | 239 (28%) | 1325 (34%) | 219 (33%) | 1008 (33%) | 0.019 |
| HF <6 months | 6116 (59%) | 1189 (58%) | 495 (58%) | 2450 (63%) | 351 (53%) | 1631 (55%) | <0.0001 |
| HF ≥6 months | 4295 (41%) | 863 (42%) | 352 (42%) | 1433 (37%) | 309 (47%) | 1338 (45%) | <0.0001 |
| HF admission in the preceding year | 3180 (29.5%) | 791 (38%) | 320 (37%) | 1617 (41%) | 255 (38%) | 1307 (43%) |  |
| Etiology |  |  |  |  |  |  |  |
| DCM | 605 (7%) | 99 (6%) | 63 (9%) | 250 (8%) | 37 (7%) | 156 (7%) | 0.0088 |
| Other/Unknown | 2888 (35%) | 550 (33%) | 244 (37%) | 1121 (36%) | 171 (33%) | 802 (34%) |  |
| Hypertension | 1665 (20%) | 371 (22%) | 116 (17%) | 573 (18%) | 113 (22%) | 492 (21%) |  |
| Ischemic Heart disease | 2664 (32%) | 543 (33%) | 212 (32%) | 986 (32%) | 163 (32%) | 760 (32%) |  |
| Heart valve disease | 476 (6%) | 86 (5%) | 31 (5%) | 192 (6%) | 29 (6%) | 138 (6%) |  |
| HF sub-type |  |  |  |  |  |  | <0.0001 |
| HFpEF | 1427 (14%) | 299 (15%) | 94 (11%) | 471 (13%) | 89 (14%) | 474 (17%) |  |
| HFmrEF | 2341 (24%) | 472 (24%) | 204 (25%) | 826 (22%) | 159 (26%) | 680 (24%) |  |
| HFrEF | 6128 (62%) | 1160 (60%) | 522 (64%) | 2429 (65%) | 371 (60%) | 1646 (59%) |  |
| NYHA III/IV | 3451 (35%) | 602 (31%) | 241 (30%) | 1425 (38%) | 179 (28%) | 1004 (35%) | <0.0001 |
| NT-BNP, median (IQR) | 2000 (906, 4060) | 1742 (759, 3718) | 1966 (88, 3700) | 2182 (1050, 4588) | 1540 (706, 3595) | 1990 (961, 3951) | <0.0001 |
| Systolic BP, mean (SD) | 127 (20) | 128 (20) | 129 (20) | 126 (21) | 127 (20) | 127 (20) | 0.0013 |
| Heart rate, median (IQR) | 70 (61, 80) | 70 (60, 80) | 70 (60, 80) | 71 (62, 81) | 70 (61, 80) | 70 (61, 80) | <0.0001 |
| eGFR, mean (SD) | 67 (21) | 67 (21) | 69 (21) | 68 (21) | 66 (21) | 64 (21) | <0.0001 |
| HB (g/L), mean (SD) | 136 (16) | 136 (16) | 138 (15) | 136 (16) | 136 (16) | 135 (17) | <0.0001 |
| BMI, median (IQR) | 27 (24, 30) | 27 (24, 30) | 27 (24, 30) | 27 (24, 30) | 27 (24, 30) | 27 (24, 31) | 0.94 |
| Potassium, mean (SD) | 4.2 (0.4) | 4.2 (0.4) | 4.2 (0.4) | 4.2 (0.4) | 4.2 (0.4) | 4.2 (0.4) | 0.042 |
| Beta blocker | 9582 (90%) | 1873 (90%) | 777 (90%) | 3614 (92%) | 605 (90%) | 2713 (89%) | 0.014 |
| RASI | 9803 (92%) | 1907 (92%) | 812 (94%) | 3674 (93%) | 631 (94%) | 2779 (91%) | 0.0076 |
| MRA | 3328 (32%) | 664 (32%) | 262 (30%) | 1260 (32%) | 203 (30%) | 939 (31%) | 0.71 |
| Digoxin | 1348 (13%) | 262 (13%) | 105 (12%) | 502 (13%) | 80 (12%) | 399 (13%) | 0.89 |
| Nitrates | 1065 (10%) | 221 (11%) | 71 (8%) | 340 (9%) | 70 (10%) | 363 (12%) | <0.0001 |
| Diuretic | 7531 (71%) | 1452 (70%) | 570 (66%) | 2805 (71%) | 468 (70%) | 2236 (74%) | <0.0001 |
| Statin | 5545 (52%) | 1104 (53%) | 478 (55%) | 2028 (51%) | 345 (51%) | 1590 (52%) | 0.46 |
| Anti Platelet | 4438 (42%) | 854 (41%) | 373 (43%) | 1627 (41%) | 293 (44%) | 1291 (43%) | 0.55 |
| Anti coagulant | 5073 (48%) | 999 (48%) | 396 (46%) | 1893 (48%) | 309 (46%) | 1476 (49%) | 0.53 |
| Revascularisation | 2997 (29%) | 588 (29%) | 252 (30%) | 1059 (27%) | 184 (28%) | 914 (31%) | 0.038 |
| Device | 1396 (13%) | 295 (14%) | 117 (14%) | 446 (11%) | 114 (17%) | 424 (14%) | <0.0001 |
| Smoking Never | 2758 (42%) | 528 (42%) | 236 (42%) | 1047 (42%) | 178 (43%) | 769 (40%) | 0.55 |
| Smoking Previous | 3110 (47%) | 589 (47%) | 270 (48%) | 1163 (47%) | 179 (44%) | 909 (47%) |  |
| Smoking current | 764 (12%) | 148 (12%) | 59 (10%) | 266 (11%) | 54 (13%) | 237 (12%) |  |
| Myocardial infarction | 2657 (33%) | 546 (34%) | 225 (34%) | 965 (32%) | 169 (34%) | 752 (33%) | 0.48 |
| Dilated cardiomyopathy | 1402 (14%) | 241 (12%) | 135 (16%) | 548 (14%) | 103 (16%) | 375 (13%) | 0.0052 |
| Valve disease | 1638 (16%) | 312 (15%) | 124 (15%) | 614 (16%) | 94 (14%) | 494 (17%) | 0.38 |
| Valve surgery | 629 (6%) | 119 (6%) | 42 (5%) | 246 (6%) | 41 (6%) | 181 (6%) | 0.63 |
| Atrial arrhythmia | 5040 (48%) | 989 (48%) | 388 (45%) | 1857 (47%) | 305 (46%) | 1501 (50%) | 0.098 |
| Hypertension | 5518 (53%) | 1089 (53%) | 449 (53%) | 1948 (50%) | 357 (54%) | 1675 (56%) | <0.0001 |
| Diabetes | 2267 (21%) | 465 (22%) | 148 (17%) | 810 (21%) | 142 (21%) | 702 (23%) | 0.0018 |
| COPD | 1448 (14%) | 296 (14%) | 114 (13%) | 493 (13%) | 72 (11%) | 473 (16%) | <0.0001 |
| Shortness of breath | 8845 (86%) | 1702 (84%) | 709 (84%) | 3345 (87%) | 560 (86%) | 2529 (86%) | 0.026 |
| Fatigue | 8661 (84%) | 1686 (84%) | 671 (80%) | 3270 (85%) | 558 (86%) | 2476 (84%) | <0.0001 |
| Pain or discomfort | 5156 (50%) | 986 (49%) | 400 (48%) | 1948 (51%) | 309 (48%) | 1513 (51%) | 0.12 |
| Anxiety or depression | 4386 (43%) | 804 (40%) | 305 (36%) | 1888 (49%) | 250 (39%) | 1139 (39%) | <0.0001 |
| Problems walking around | 4278 (41%) | 789 (39%) | 283 (34%) | 1615 (42%) | 232 (36%) | 1359 (46%) | <0.0001 |
| Problems with ADL | 3266 (32%) | 537 (27%) | 218 (26%) | 1385 (36%) | 163 (25%) | 963 (33%) | <0.0001 |
| Problems with self care | 989 (10%) | 181 (9%) | 47 (6%) | 358 (9%) | 51 (8%) | 352 (12%) | <0.0001 |
| All results reported as n (%) unless otherwise stated. HF, heart failure; HFpEF, heart failure with preserved ejection fraction; HFmrEF, heart failure with mildy-reduced ejection fraction; HFrEF, heart failure with ; reduced ejection fraction; NYHA, New York Heart Association; NT-BNP, NT-proB-type Natriuretic Peptide; eGFR, estimated glomerular filtration rate; HB, hemoglobin; BMI, body mass index; RASI, renin-angiotensin system inhibitors (comprising Angiotensin-converting enzyme inhibitors, Angiotensin receptor blockers and angiotensin Receptor-Neprilysin Inhibitor); MRA, Mineralocorticoid Receptor Antagonists; COPD, chronic obstructive pulmonary disease; ADL, activities of daily living. | | | | | | | |

# **S4 Table: Change in Euro-Qol Visual Analogue Scale (EQ5D-vas) by population groups (sex, age and HF sub-type)**

|  | Total | Male | Female | Youngest (≤60 years) | Oldest (>85 years) | HFpEF | HFmrEF | HFrEF |
| --- | --- | --- | --- | --- | --- | --- | --- | --- |
|  | N=10,603 | N=7,128 | N=3,475 | N=1,623 | N=835 | N=1,427 | N=2,341 | N=6,128 |
| Best: no change | 2,081 (20%) | 1,397 (20%) | 684 (20%) | 311 (19%) | 162 (19%) | 299 (21%) | 472 (20%) | 1,160 (19%) |
| Some improvement: 5 to 9 points | 865 (8%) | 626 (9%) | 239 (7%) | 145 (9%) | 41 (5%) | 94 (7%) | 204 (9%) | 522 (9%) |
| Considerable improvement: >10 points | 3,944 (37%) | 2,619 (37%) | 1,325 (38%) | 714 (44%) | 259 (31%) | 471 (33%) | 826 (35%) | 2,429 (40%) |
| Some worsening: 5 to 9 points | 672 (6%) | 453 (6%) | 219 (6%) | 88 (5%) | 50 (6%) | 89 (6%) | 159 (7%) | 371 (6%) |
| Considerable worsening: >10 points | 3,041 (29%) | 2,033 (29%) | 1,008 (29%) | 365 (22%) | 323 (39%) | 474 (33%) | 680 (29%) | 1,646 (27%) |

Data are presented as n (%). HFpEF, heart failure with preserved ejection fraction; HFmrEF, heart failure with mildy-reduced ejection fraction; HFrEF, heart failure with reduced ejection fraction. Differences by sex (p=0.0188), age (p<0.0001), HF subtype (p<0.0001).

| S5 Table: Interaction between EQ5D-vas and age | | | | | |  |
| --- | --- | --- | --- | --- | --- | --- |
|  | **1^st^ hospitalization for any cause** | **1^st^ hospitalization for HF** | |  |  |  |
| EQ5D-VAS at baseline | Adjusted HR (95 CI) | Adjusted HR (95 CI) | |  |  |  |
| ≤ 60 years |  |  | |  |  |  |
| 76-100 (best) | 1.0 | 1.0 | |  |  |  |
| 51-75 | 1.10 (0.98 - 1.24) | 1.06 (0.84 - 1.34) | |  |  |  |
| 26-50 | 1.20 (1.05 - 1.37) | 1.23 (0.96 - 1.58) | |  |  |  |
| 0-25 (worst) | 1.39 (1.12 - 1.72) | 1.19 (0.82 - 1.72) | |  |  |  |
| 61-65 years |  |  | |  |  |  |
| 76-100 (best) | 1.0 | 1.0 | |  |  |  |
| 51-75 | 1.19 (1.03 - 1.37) | 0.99 (0.76 - 1.29) | |  |  |  |
| 26-50 | 1.51 (1.28 - 1.77) | 1.52 (1.15 - 2.02) | |  |  |  |
| 0-25 (worst) | 1.62 (1.22 - 2.16) | 1.01 (0.64 - 1.61) | |  |  |  |
| 66-70 years |  |  | |  |  |  |
| 76-100 (best) | 1.0 | 1.0 | |  |  |  |
| 51-75 | 1.01 (0.90 - 1.13) | 0.89 (0.73 - 1.08) | |  |  |  |
| 26-50 | 1.26 (1.10 - 1.44) | 1.19 (0.95 - 1.50) | |  |  |  |
| 0-25 (worst) | 1.07 (0.84 - 1.36) | 0.99 (0.68 - 1.44) | |  |  |  |
| 71-75 years |  |  | |  |  |  |
| 76-100 (best) | 1.0 | 1.0 | |  |  |  |
| 51-75 | 1.14 (1.03 - 1.25) | 1.08 (0.91 - 1.28) | |  |  |  |
| 26-50 | 1.32 (1.17 - 1.47) | 1.30 (1.08 - 1.58) | |  |  |  |
| 0-25 (worst) | 1.56 (1.26 - 1.92) | 1.22 (0.88 - 1.69) | |  |  |  |
| 76-80 years |  |  | |  |  |  |
| 76-100 (best) | 1.0 | 1.0 | |  |  |  |
| 51-75 | 1.13 (1.03 - 1.25) | 1.06 (0.89 - 1.25) | |  |  |  |
| 26-50 | 1.37 (1.23 - 1.53) | 1.31 (1.09 - 1.57) | |  |  |  |
| 0-25 (worst) | 1.49 (1.22 - 1.81) | 1.46 (1.07 - 1.98) | |  |  |  |
| >81-85 years |  |  | |  |  |  |
| 76-100 (best) | 1.0 | 1.0 | |  |  |  |
| 51-75 | 1.03 (0.93 - 1.14) | 0.92 (0.78 - 1.09) | |  |  |  |
| 26-50 | 1.15 (1.02 - 1.29) | | 1.04 (0.86 - 1.25) |  |  |  |
| 0-25 (worst) | 1.34 (1.09 - 1.64) | 1.42 (1.07 - 1.90) | |  |  |  |
| >85 years |  |  | |  |  |  |
| 76-100 (best) | 1.0 | 1.0 | |  |  |  |
| 51-75 | 1.06 (0.93 - 1.20) | 0.98 (0.81 - 1.19) | |  |  |  |
| 26-50 | 1.11 (0.97 - 1.27) | 1.05 (0.85 - 1.29) | |  |  |  |
| 0-25 (worst) | 1.18 (0.92 - 1.51) | 0.97 (0.66 - 1.43) | |  |  |  |
| HF, heart failure; EQ5D-VAS, Euro-QoL-5D visual analogue scale; pts, points  *See table 3 in main results for adjustment variables. | | | | | | |

| S6 Table: Interaction between EQ5D-vas change and sex | | | | |
| --- | --- | --- | --- | --- |
|  | **1^st^ hospitalization for any cause** | **1^st^ hospitalization for HF** |  |  |
| EQ5D-VAS change from baseline | Adjusted HR (95 CI) | Adjusted HR (95 CI) |  |  |
| Men |  |  |  |  |
| No change | 1.0 | 1.0 |  |  |
| Improve 5-9 pts | 0.90 (0.79 - 1.02) | 0.93 (0.74 - 1.17) |  |  |
| Improve >10 pts | 0.77 (0.70 - 0.84) | 0.75 (0.64 - 0.89) |  |  |
| Worse 5-9 pts | 0.99 (0.86 - 1.14) | 1.01 (0.80 - 1.29) |  |  |
| Worse >10 pts | 1.30 (1.19 - 1.42) | 1.30 (1.11 - 1.51) |  |  |
| Female |  |  |  |  |
| No change | 1.0 | 1.0 |  |  |
| Improve 5-9 pts | 0.96 (0.79 - 1.17) | 1.04 (0.72 - 1.49) |  |  |
| Improve >10 pts | 0.71 (0.63 - 0.81) | 0.60 (0.47 - 0.77) |  |  |
| Worse 5-9 pts | 1.11 (0.91 - 1.35) | 0.76 (0.52 - 1.11) |  |  |
| Worse >10 pts | 1.16 (1.02 - 1.31) | 1.10 (0.88 - 1.39) |  |  |
| HF, heart failure; EQ5D-VAS, Euro-QoL-5D visual analogue scale; pts, points  *See table 3 in main results for adjustment variables. | | | | |

| S7 Table: Time dependent association between lowest EQ5D-vas (0-25) and outcomes | | | | |  |
| --- | --- | --- | --- | --- | --- |
| Months in follow-up | Any-cause hospitalisation | HF hospitalisation | Death | Graphs showing the HR (y axis) plotted against month during follow-up (x axis)  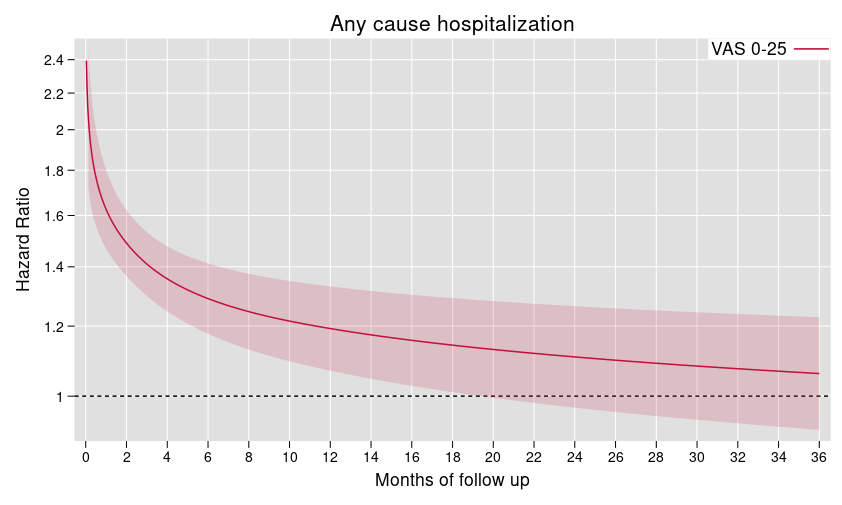  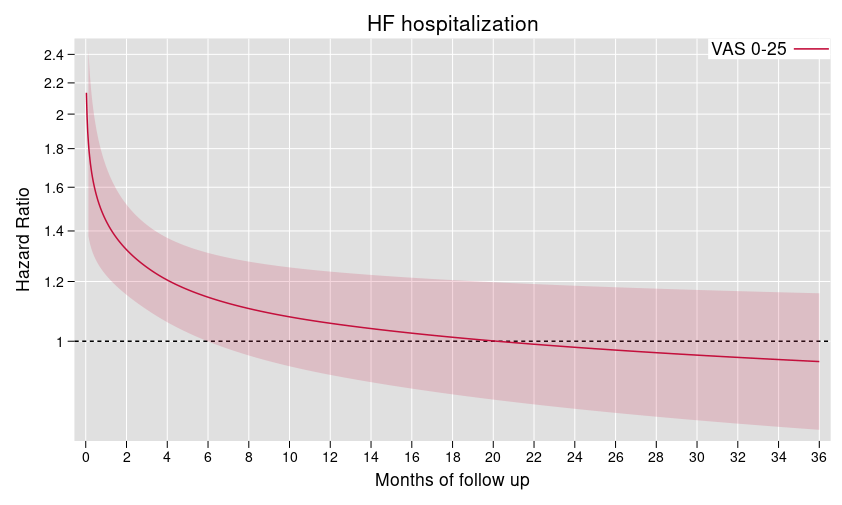  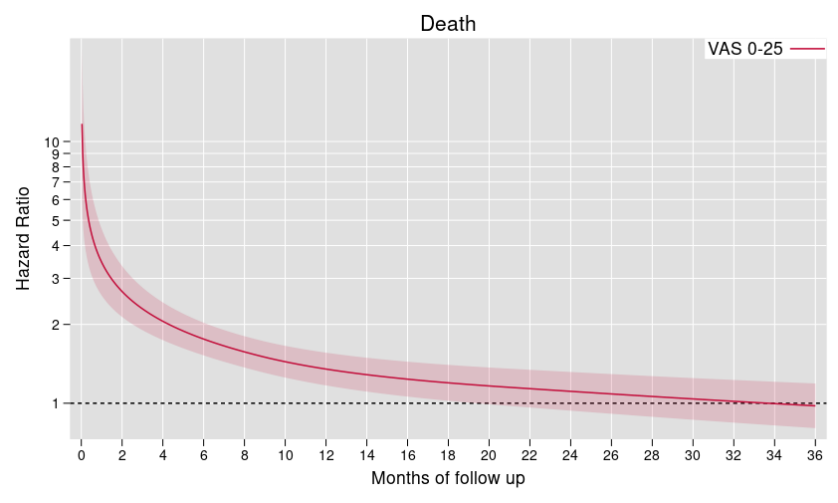 | |
|  | HR (p<0.001) | P=0.002 | (p<0.001) |  |  |
| 1 | 1.63 (1.47 ,1.80) | 1.44 (1.22 ,1.70) | 3.46 (2.58 ,4.63) |  |  |
| 2 | 1.49 (1.37 ,1.62) | 1.32 (1.15 ,1.52) | 2.67 (2.14 ,3.34) |  |  |
| 3 | 1.41 (1.30 ,1.53) | 1.25 (1.10 ,1.43) | 2.29 (1.90 ,2.77) |  |  |
| 4 | 1.36 (1.25 ,1.48) | 1.21 (1.06 ,1.37) | 2.06 (1.74 ,2.43) |  |  |
| 5 | 1.32 (1.21 ,1.44) | 1.17 (1.03 ,1.33) | 1.89 (1.62 ,2.20) |  |  |
| 6 | 1.29 (1.18 ,1.41) | 1.14 (1.00 ,1.31) | 1.76 (1.52 ,2.03) |  |  |
| 7 | 1.27 (1.15 ,1.39) | 1.12 (0.98 ,1.29) | 1.66 (1.44 ,1.90) |  |  |
| 8 | 1.25 (1.13 ,1.38) | 1.10 (0.96 ,1.28) | 1.57 (1.37 ,1.80) |  |  |
| 9 | 1.23 (1.11 ,1.36) | 1.09 (0.94 ,1.26) | 1.50 (1.31 ,1.72) |  |  |
| 10 | 1.22 (1.10 ,1.35) | 1.08 (0.93 ,1.25) | 1.44 (1.25 ,1.66) |  |  |
| 11 | 1.20 (1.08 ,1.34) | 1.07 (0.91 ,1.24) | 1.39 (1.21 ,1.60) |  |  |
| 12 | 1.19 (1.07 ,1.33) | 1.06 (0.90 ,1.24) | 1.35 (1.17 ,1.56) |  |  |
| 13 | 1.18 (1.06 ,1.32) | 1.05 (0.89 ,1.23) | 1.32 (1.14 ,1.52) |  |  |
| 14 | 1.17 (1.05 ,1.31) | 1.04 (0.88 ,1.22) | 1.29 (1.11 ,1.49) |  |  |
| 15 | 1.16 (1.04 ,1.31) | 1.03 (0.87 ,1.22) | 1.26 (1.08 ,1.46) |  |  |
| 16 | 1.16 (1.03 ,1.30) | 1.02 (0.87 ,1.21) | 1.24 (1.06 ,1.44) |  |  |
| 17 | 1.15 (1.02 ,1.30) | 1.02 (0.86 ,1.21) | 1.22 (1.04 ,1.42) |  |  |
| 18 | 1.14 (1.01 ,1.29) | 1.01 (0.85 ,1.21) | 1.20 (1.02 ,1.40) |  |  |
| 19 | 1.14 (1.00 ,1.29) | 1.01 (0.84 ,1.20) | 1.18 (1.01 ,1.38) |  |  |
| 20 | 1.13 (1.00 ,1.28) | 1.00 (0.84 ,1.20) | 1.16 (0.99 ,1.37) |  |  |
| 21 | 1.12 (0.99 ,1.28) | 1.00 (0.83 ,1.19) | 1.15 (0.98 ,1.35) |  |  |
| 22 | 1.12 (0.98 ,1.27) | 0.99 (0.82 ,1.19) | 1.14 (0.96 ,1.34) |  |  |
| 23 | 1.11 (0.98 ,1.27) | 0.99 (0.82 ,1.19) | 1.12 (0.95 ,1.33) |  |  |
| 24 | 1.11 (0.97 ,1.26) | 0.98 (0.81 ,1.18) | 1.11 (0.94 ,1.32) |  |  |
| 25 | 1.10 (0.96 ,1.26) | 0.98 (0.81 ,1.18) | 1.10 (0.92 ,1.30) |  |  |
| 26 | 1.10 (0.96 ,1.26) | 0.97 (0.80 ,1.18) | 1.09 (0.91 ,1.29) |  |  |
| 27 | 1.09 (0.95 ,1.25) | 0.97 (0.80 ,1.18) | 1.07 (0.90 ,1.28) |  |  |
| 28 | 1.09 (0.95 ,1.25) | 0.97 (0.79 ,1.17) | 1.06 (0.89 ,1.27) |  |  |
| 29 | 1.09 (0.94 ,1.25) | 0.96 (0.79 ,1.17) | 1.05 (0.88 ,1.26) |  |  |
| 30 | 1.08 (0.94 ,1.24) | 0.96 (0.79 ,1.17) | 1.04 (0.86 ,1.25) |  |  |
| 31 | 1.08 (0.94 ,1.24) | 0.96 (0.78 ,1.17) | 1.03 (0.85 ,1.24) |  |  |
| 32 | 1.07 (0.93 ,1.24) | 0.95 (0.78 ,1.17) | 1.02 (0.84 ,1.23) |  |  |
| 33 | 1.07 (0.93 ,1.24) | 0.95 (0.77 ,1.16) | 1.01 (0.83 ,1.22) |  |  |
| 34 | 1.07 (0.92 ,1.23) | 0.95 (0.77 ,1.16) | 1.00 (0.82 ,1.21) |  |  |
| 35 | 1.06 (0.92 ,1.23) | 0.94 (0.77 ,1.16) | 0.99 (0.81 ,1.20) |  |  |
| 36 | 1.06 (0.92 ,1.23) | 0.94 (0.76 ,1.16) | 0.98 (0.80 ,1.19) |  |  |
|  |  |  |  |  |  |
| HF, heart failure; EQ5D-VAS, Euro-QoL-5D visual analogue scale; pts, points. EQ5D-vas exposures added as a time dependant covariate with df=1. *See table 3 in main results for adjustment variables | | | | | |

| S8 Table: Time dependent association between considerable worsening of EQ5D-vas (>10 points) and outcomes | | | |
| --- | --- | --- | --- |
| Months in follow-up | Any-cause hospitalisation | HF hospitalisation | Graphs showing the HR (y axis) plotted against month during follow-up (x axis) |
|  | P=0.059 | P=0.018 |  |
| 1 | 1.38 (1.23 ,1.56) | 1.63 (1.28 ,2.07) | **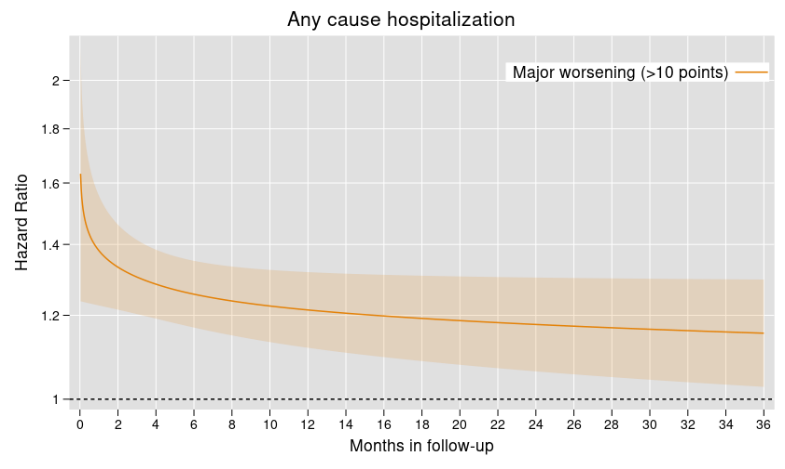**  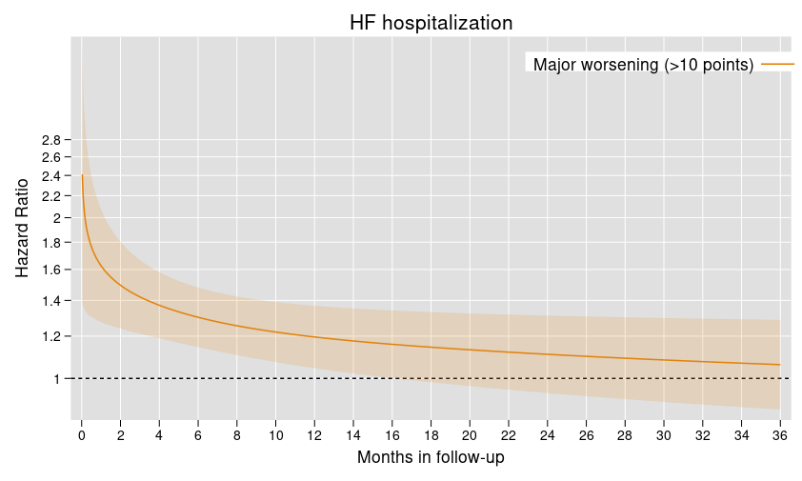 |
| 2 | 1.33 (1.21 ,1.46) | 1.49 (1.24 ,1.80) |  |
| 3 | 1.30 (1.20 ,1.41) | 1.42 (1.21 ,1.67) |  |
| 4 | 1.28 (1.19 ,1.38) | 1.37 (1.19 ,1.58) |  |
| 5 | 1.27 (1.18 ,1.36) | 1.33 (1.17 ,1.52) |  |
| 6 | 1.26 (1.17 ,1.35) | 1.30 (1.14 ,1.48) |  |
| 7 | 1.25 (1.16 ,1.34) | 1.28 (1.12 ,1.45) |  |
| 8 | 1.24 (1.15 ,1.33) | 1.25 (1.11 ,1.42) |  |
| 9 | 1.23 (1.14 ,1.33) | 1.24 (1.09 ,1.41) |  |
| 10 | 1.22 (1.13 ,1.32) | 1.22 (1.07 ,1.39) |  |
| 11 | 1.22 (1.12 ,1.32) | 1.21 (1.06 ,1.38) |  |
| 12 | 1.21 (1.12 ,1.32) | 1.20 (1.05 ,1.37) |  |
| 13 | 1.21 (1.11 ,1.32) | 1.18 (1.03 ,1.36) |  |
| 14 | 1.21 (1.11 ,1.31) | 1.18 (1.02 ,1.35) |  |
| 15 | 1.20 (1.10 ,1.31) | 1.17 (1.01 ,1.35) |  |
| 16 | 1.20 (1.10 ,1.31) | 1.16 (1.00 ,1.34) |  |
| 17 | 1.20 (1.09 ,1.31) | 1.15 (0.99 ,1.34) |  |
| 18 | 1.19 (1.09 ,1.31) | 1.14 (0.98 ,1.33) |  |
| 19 | 1.19 (1.08 ,1.31) | 1.14 (0.98 ,1.33) |  |
| 20 | 1.19 (1.08 ,1.31) | 1.13 (0.97 ,1.32) |  |
| 21 | 1.18 (1.07 ,1.30) | 1.13 (0.96 ,1.32) |  |
| 22 | 1.18 (1.07 ,1.30) | 1.12 (0.95 ,1.32) |  |
| 23 | 1.18 (1.07 ,1.30) | 1.11 (0.95 ,1.31) |  |
| 24 | 1.18 (1.06 ,1.30) | 1.11 (0.94 ,1.31) |  |
| 25 | 1.17 (1.06 ,1.30) | 1.10 (0.93 ,1.31) |  |
| 26 | 1.17 (1.06 ,1.30) | 1.10 (0.93 ,1.31) |  |
| 27 | 1.17 (1.05 ,1.30) | 1.10 (0.92 ,1.30) |  |
| 28 | 1.17 (1.05 ,1.30) | 1.09 (0.91 ,1.30) |  |
| 29 | 1.17 (1.05 ,1.30) | 1.09 (0.91 ,1.30) |  |
| 30 | 1.16 (1.04 ,1.30) | 1.08 (0.90 ,1.30) |  |
| 31 | 1.16 (1.04 ,1.30) | 1.08 (0.90 ,1.30) |  |
| 32 | 1.16 (1.04 ,1.30) | 1.08 (0.89 ,1.29) |  |
| 33 | 1.16 (1.03 ,1.30) | 1.07 (0.89 ,1.29) |  |
| 34 | 1.16 (1.03 ,1.30) | 1.07 (0.88 ,1.29) |  |
| 35 | 1.16 (1.03 ,1.30) | 1.06 (0.88 ,1.29) |  |
| 36 | 1.15 (1.03 ,1.30) | 1.06 (0.87 ,1.29) |  |
| HF, heart failure; EQ5D-vas, euro-qol-5d visual analogue scale; pts, points. EQ5D-vas exposures added as a time dependant covariate with df=1. *See table 3 in main results for adjustment variables | | | |

|  | | | | | | | | | |  |  |  | | |  |
| --- | --- | --- | --- | --- | --- | --- | --- | --- | --- | --- | --- | --- | --- | --- | --- |
| S9 Table: Sensitivity analysis, removing patients with an admission prior to the change analysis. | | | | | | | | | |  |  |  | | |  |
|  |  | | | | | | |  | | |  |  |  |  |  |
|  | **1^st^ hospitalization for any cause** | | | | **1^st^ hospitalization for HF** | | | | **Death** | | | | |  |  |
|  | Adjusted (all) | | Adjusted (no prior HF admission in 1 yr) | | Adjusted (all) | | Adjusted (no prior HF admission in 1 yr) | | Adjusted (all) | Adjusted (no prior HF admission in 1 yr) | | | |  |  |
|  | HR (95 CI) | | | | HR (95 CI) | | | | HR (95 CI) | | | | |  |  |
| EQ5D-vas at baseline (n=13,907) | | | | |  | |  | |  |  | | | |  |  |
| 76-100 (best) | 1.0 | | 1.0 | | 1.0 | | 1.0 | | 1.0 | 1.0 | | | |  |  |
| 51-75 | 1.09 (1.04,1.14) | | 1.13 (1.06,1.19) | | 1.01 (0.94,1.09) | | 1.14 (1.02,1.28) | | 1.15 (1.06,1.25) | 1.31 (1.16,1.49) | | | |  |  |
| 26-50 | 1.27 (1.21,1.33) | | 1.30 (1.22,1.39) | | 1.22 (1.12,1.31) | | 1.39 (1.23,1.58) | | 1.37 (1.26,1.49) | 1.48 (1.29,1.70) | | | |  |  |
| 0-25 (worst) | 1.39 (1.28,1.51) | | 1.42 (1.26,1.61) | | 1.22 (1.07,1.38) | | 1.25 (1.00,1.57) | | 1.50 (1.31,1.72) | 1.51 (1.20,1.90) | | | |  |  |
| EQ5D-vas change over 1 year (n=6,262) | | | |  | | | | |  | | | |  |  |  |
| No change | 1.00 (Ref) | 1.00 (Ref) | | 1.00 (Ref) | | 1.00 (Ref) | | | 1.00 (Ref) | 1.00 (Ref) | | |  |  |  |
| Improve 5-9 pts | 0.91 (0.82,1.01) | 0.85 (0.73,0.98) | | 0.95 (0.78,1.15) | | 0.75 (0.56,1.01) | | | 0.84 (0.68,1.03) | 0.70 (0.52,0.95) | | |  |  |  |
| Improve >10 pts | 0.75 (0.70,0.81) | 0.76 (0.69,0.83) | | 0.71 (0.62,0.81) | | 0.66 (0.54,0.80) | | | 0.71 (0.62,0.81) | 0.66 (0.55,0.80) | | |  |  |  |
| Worse 5-9 pts | 1.04 (0.92,1.16) | 0.97 (0.83,1.13) | | 0.93 (0.76,1.14) | | 0.91 (0.68,1.23) | | | 0.94 (0.77,1.16) | 0.87 (0.64,1.18) | | |  |  |  |
| Worse >10 pts | 1.25 (1.16,1.35) | 1.24 (1.13,1.36) | | 1.25 (1.10,1.41) | | 1.18 (0.99,1.42) | | | 1.37 (1.20,1.55) | 1.36 (1.14,1.63) | | |  |  |  |
| HF, heart failure; EQ5D-vas, Euro-QoL visual analogue scale; pts, points  All models adjusted for age, sex, HF type, HF duration, etiology, NYHA, NTpro-BNP, systolic blood pressure (SBP), heart rate (HR), estimated glomerular filtration rate (eGFR), hemoglobin (HB), BMI, potassium, BB, RASI (comprising Angiotensin-converting enzyme inhibitors, Angiotensin receptor blockers and angiotensin Receptor-Neprilysin Inhibitor), MRA, digoxin, nitrates, diuretic, anti coagulant, anti-platelet, revascularisation, device, smoking, myocardial infarction (MI), dilated cardiomyopathy, valve disease, valve surgery, atrial arrhythmia , hypertension, diabetes, lung disease.  *EQ5D-VAS change models also adjusted for baseline EQ5D-VAS. | | | | | | | | | | | | | |  |  |

| S10 Table: Sensitivity analysis using complete cases. | | | | | | | | | | | |  |  | |  |
| --- | --- | --- | --- | --- | --- | --- | --- | --- | --- | --- | --- | --- | --- | --- | --- |
|  |  | | | | | | |  |  |  |  |  |  |  |  |
|  | | **1^st^ hospitalization for HF** | **1^st^ hospitalization for any cause** | | **Death** |  | |  |  |  |  |  |  |  |  |
| EQ5D-VAS at baseline | | Adjusted | Adjusted | Adjusted | | |  |  |  |  |  |  |  |  |  |
| (n=5,565) | |  |  |  |  |  |  |  |  |  |  |  |  |  |  |
| 76-100 (best) | | 1.0 | 1.0 | 1.0 | | |  |  |  |  |  |  |  |  |  |
| 51-75 | | 1.15 (0.97,1.36) | 1.11 (1.01,1.22) | 1.23 (0.99,1.53) | | |  |  |  |  |  |  |  |  |  |
| 26-50 | | 1.38 (1.15,1.66) | 1.33 (1.20,1.47) | 1.44 (1.15,1.81) | | |  |  |  |  |  |  |  |  |  |
| 0-25 (worst) | | 1.49 (1.13,1.98) | 1.60 (1.34,1.91) | 1.57 (1.11,2.22) | | |  |  |  |  |  |  |  |  |  |
| Adjusted*  EQ5D-VAS change  over 1 year (n=1,690) | | | Adjusted* | Adjusted* | | |  |  |  |  |  |  |  |  |  |
| No change | | 1.00 (Ref) | 1.00 (Ref) | 1.00 (Ref) | | |  |  |  |  |  |  |  |  |  |
| Improve 5-9 pts | | 0.80 (0.43,1.50) | 0.90 (0.65,1.25) | 0.92 (0.46,1.83) | | |  |  |  |  |  |  |  |  |  |
| Improve >10 pts | | 0.63 (0.42,0.95) | 0.84 (0.68,1.05) | 0.69 (0.44,1.09) | | |  |  |  |  |  |  |  |  |  |
| Worse 5-9 pts | | 0.82 (0.42,1.59) | 1.35 (0.96,1.91) | 1.41 (0.72,2.77) | | |  |  |  |  |  |  |  |  |  |
| Worse >10 pts | | 1.36 (0.93,2.00) | 1.46 (1.17,1.81) | 1.48 (0.96,2.29) | | |  |  |  |  |  |  |  |  |  |
| HF, heart failure; EQ5D-VAS, Euro-QoL visual analogue scale; pts, points  All models adjusted for age, sex, HF type, HF duration, previous HD admission in 1 year, etiology, NYHA, NTpro-BNP, systolic blood pressure (SBP), heart rate (HR), estimated glomerular filtration rate (eGFR), hemoglobin (HB), BMI, potassium, BB, RASI, MRA, digoxin, nitrates, diuretic, anti coagulant, anti-platelet, revascularisation, device, smoking, myocardial infarction (MI), dilated cardiomyopathy, valve disease, valve surgery, atrial arrhythmia, hypertension, diabetes, chronic obstructive pulmonary disease.  *EQ5D-VAS change models also adjusted for baseline EQ5D-VAS. | | | | | | | | | | | | | |  |  |

# **S1 Figure: Probability of admission by baseline EQ5D-vas (fitted versus non-parametric Kaplan Meier curves)**

**Hospitalization for HF**

)

**Hospitalization for any cause**


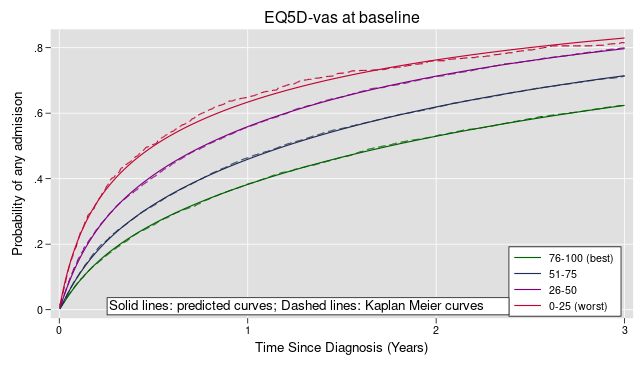

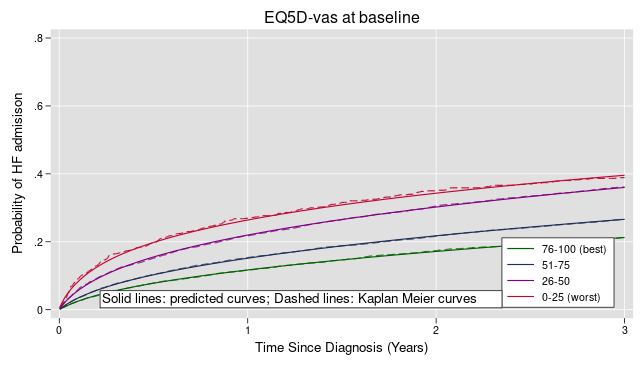


|  | Number at risk | | | | | | |
| --- | --- | --- | --- | --- | --- | --- | --- |
|  | 0M | 6M | 12M | 18M | 24M | 30M | 36M |
| 76-100 | 6794 | 5005 | 3659 | 2808 | 2210 | 1766 | 1443 |
| 51-75 | 9751 | 6577 | 4586 | 3475 | 2693 | 2096 | 1673 |
| 26-50 | 5971 | 3498 | 2272 | 1605 | 1203 | 883 | 680 |
| 0-25 | 1017 | 476 | 300 | 212 | 155 | 120 | 94 |

|  | Number at risk | | | | | | |
| --- | --- | --- | --- | --- | --- | --- | --- |
|  | 0M | 6M | 12M | 18M | 24M | 30M | 36M |
| 76-100 | 6794 | 6149 | 5140 | 4381 | 3764 | 3277 | 2846 |
| 51-75 | 9751 | 8484 | 6997 | 6004 | 5155 | 4429 | 3856 |
| 26-50 | 5971 | 4848 | 3836 | 3120 | 2595 | 2139 | 1836 |
| 0-25 | 1017 | 748 | 574 | 461 | 373 | 321 | 276 |

# Probability curves, for each category of EQ5D-VAS exposure estimated using the fitted model (solid line) and non-parametric Kaplan Meier curves (dashed line). Tables show the number at risk for each category of exposure during follow-up.

# **S2 Figure: Probability of admission by EQ5D-vas change (fitted versus non-parametric Kaplan Meier curves)**

**Hospitalization for HF**

)

**Hospitalization for any cause**


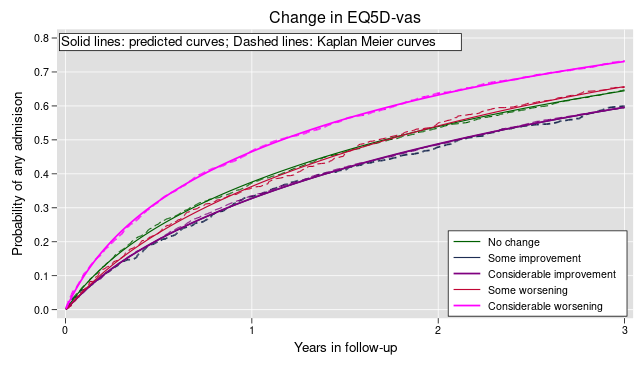

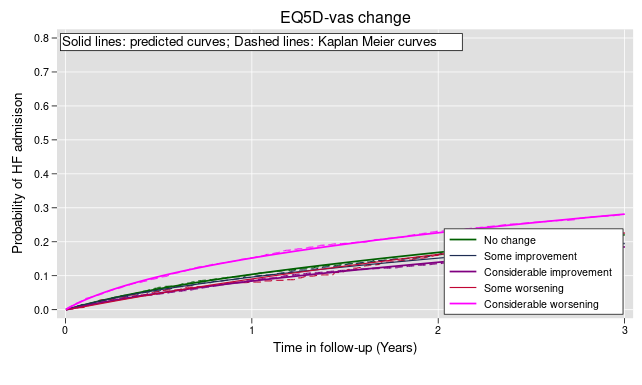


|  | | Number at risk | | | | | | | | | | | | | |  |
| --- | --- | --- | --- | --- | --- | --- | --- | --- | --- | --- | --- | --- | --- | --- | --- | --- |
|  | 0M | | 6M | 12M | 18M | 24M | 30M | 36M | 0M | 6M | 12M | 18M | 24M | 30M | 36M |  |
| no change | | 2082 | | 1441 | 1145 | 940 | 785 | 657 | 529 | 2082 | 1783 | 1595 | 1449 | 1314 | 1183 | 1041 |
| Some improvement | | 869 | | 649 | 513 | 438 | 371 | 315 | 259 | 869 | 758 | 677 | 626 | 574 | 532 | 492 |
| Considerable improve. | | 3938 | | 2957 | 2380 | 2006 | 1717 | 1459 | 1252 | 3938 | 3518 | 3171 | 2929 | 2722 | 2505 | 2280 |
| Some worsening | | 677 | | 484 | 385 | 315 | 251 | 209 | 171 | 677 | 579 | 538 | 490 | 441 | 398 | 344 |
| Considerable worse. | | 3037 | | 1946 | 1460 | 1164 | 926 | 779 | 629 | 3037 | 2518 | 2208 | 1964 | 1752 | 1606 | 1415 |

# Probability curves, for each category of EQ5D-VAS change exposure estimated using the fitted model (solid line) and non-parametric Kaplan Meier curves (dashed line). Tables show the number at risk for each category of exposure during follow-up.

# **S3 Figure: Survival curves for EQ5D-vas exposures (fitted versus non-parametric Kaplan Meier curves)**

b)


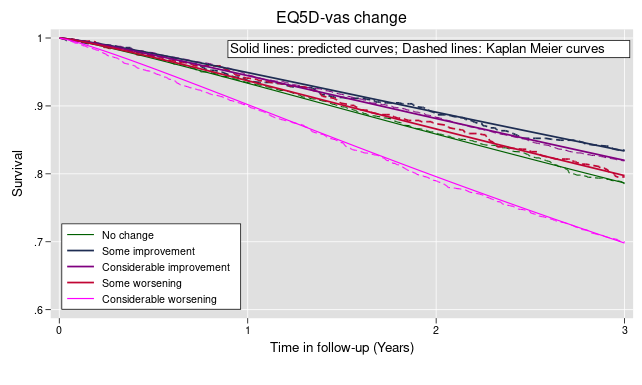

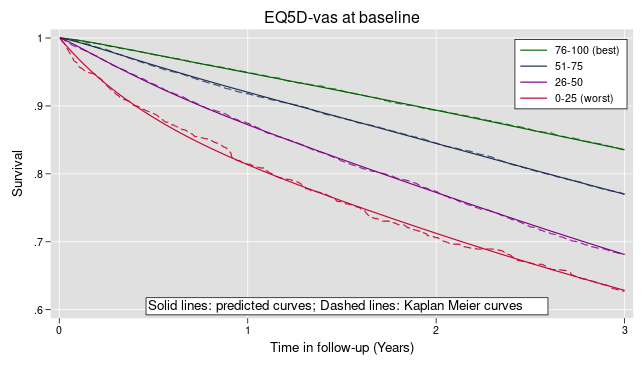


a)

|  | Number at risk | | | | | | |
| --- | --- | --- | --- | --- | --- | --- | --- |
|  | 0M | 6M | 12M | 18M | 24M | 30M | 36M |
| 76-100 | 6794 | 6627 | 5741 | 5024 | 4407 | 3881 | 3415 |
| 51-75 | 9751 | 9347 | 8057 | 7119 | 6251 | 5462 | 4823 |
| 26-50 | 5971 | 5566 | 4670 | 3963 | 3430 | 2912 | 2536 |
| 0-25 | 1017 | 904 | 738 | 616 | 513 | 443 | 387 |

|  | NUMBER AT RISK | | | | | | |
| --- | --- | --- | --- | --- | --- | --- | --- |
|  | 0M | 6M | 12M | 18M | 24M | 30M | 36M |
| no change | 2082 | 1900 | 1743 | 1617 | 1491 | 1383 | 1238 |
| Some improvement | 869 | 795 | 735 | 694 | 655 | 610 | 569 |
| Considerable improve. | 3938 | 3669 | 3418 | 3218 | 3119 | 2831 | 2607 |
| Some worsening | 677 | 608 | 573 | 536 | 501 | 464 | 415 |
| Considerable worse. | 3037 | 2736 | 2513 | 2310 | 2094 | 1934 | 1724 |

# Survival curves, for each category of EQ5D-VAS exposure estimated using the fitted model (solid line) and non-parametric Kaplan Meier curves (dashed line). Tables show the number at risk for each category of exposure during follow-up.

# **Supplementary references (30-54):**

30. Ponikowski et al, 2016 ESC Guidelines for the diagnosis and treatment of acute and chronic heart failure: The Task Force for the diagnosis and treatment of acute and chronic heart failure of the European Society of Cardiology (ESC) Developed with the special contribution of the Heart Failure Association (HFA) of the ESC, European Heart Journal, Volume 37, Issue 27, 14 July 2016, Pages 2129–2200, https://doi.org/10.1093/eurheartj/ehw128

31. Rubin DB. Multiple Imputation for Nonresponse in Surveys. New York: Wiley. New York: Wiley; 1987.

32. Royston P, Parmar MKB. Flexible parametric proportional-hazards and proportional-odds models for censored survival data, with application to prognostic modelling and estimation of treatment effects. Statist Med. 2002;21(15):2175-97.

33. Alla F, Briancon S, Guillemin F, Juilliere Y, Mertes P-, Villemot J-, et al. Self-rating of quality of life provides additional prognostic information in heart failure. Insights into the EPICAL study. European Journal of Heart Failure. 2002;4(3):337-43.

34. Heidenreich PA, Spertus JA, Jones PG, Weintraub WS, Rumsfeld JS, Rathore SS, et al. Health status identifies heart failure outpatients at risk for hospitalization or death. J Am Coll Cardiol. 2006;47(4):752-6.

35. Greene SJ, Butler J, Spertus JA, Hellkamp AS, Vaduganathan M, DeVore AD, et al. Comparison of New York Heart Association Class and Patient-Reported Outcomes for Heart Failure With Reduced Ejection Fraction. JAMA Cardiol. 2021;6(5):522-31.

36. Johansson I, Joseph P, Balasubramanian K, McMurray JJV, Lund LH, Ezekowitz JA, et al. Health-Related Quality of Life and Mortality in Heart Failure: The Global Congestive Heart Failure Study of 23 000 Patients From 40 Countries. Circulation. 2021;143(22):2129-42.

37. Kosiborod M, Soto GE, Jones PG, Krumholz HM, Weintraub WS, Deedwania P, Spertus JA. Identifying heart failure patients at high risk for near-term cardiovascular events with serial health status assessments. Circulation. 2007 Apr 17;115(15):1975-81.

38. Moser DK, Yamokoski L, Sun JL, et al; Escape Investigators. Improvement in health-related quality of life after hospitalization predicts event-free survival in patients with advanced heart failure. J Card Fail. 2009 Nov;15(9):763-9. doi: 10.1016/j.cardfail.2009.05.003. Epub 2009 Jun 25. PMID: 19879462; PMCID: PMC2772831.

39. Johansson I, Joseph P, Balasubramanian K, McMurray JJV, Lund LH, Ezekowitz JA, et al. Health-Related Quality of Life and Mortality in Heart Failure: The Global Congestive Heart Failure Study of 23 000 Patients From 40 Countries. Circulation. 2021;143(22):2129-

40. Moradi M, Daneshi F, Behzadmehr R, Rafiemanesh H, Bouya S, Raeisi M. Quality of life of chronic heart failure patients: a systematic review and meta-analysis. Heart Fail Rev. 2020 Nov;25(6):993-1006.

41. Truby LK, O'Connor C, Fiuzat M, Stebbins A, Coles A, Patel CB, et al. Sex Differences in Quality of Life and Clinical Outcomes in Patients With Advanced Heart Failure: Insights From the PAL-HF Trial. Circulation.Heart failure. 2020;13(4):e006134.

42. Fonseca AF, Lahoz R, Proudfoot C, Corda S, Loefroth E, Jackson J, et al. Burden and Quality of Life Among Female and Male Patients with Heart Failure in Europe: A Real-World Cross-Sectional Study. Patient Prefer Adherence. 2021 Jul 30;15:1693-706.

43. Blumer V, Greene SJ, Wu A, Butler J, Ezekowitz JA, Lindenfeld J, et al. Sex Differences in Clinical Course and Patient-Reported Outcomes Among Patients Hospitalized for Heart Failure. JACC: Heart Failure. 2021;9(5):336-45.

44. Hou N, Chui MA, Eckert GJ, Oldridge NB, Murray MD, Bennett SJ. Relationship of age and sex to health-related quality of life in patients with heart failure. Am J Crit Care. 2004 Mar;13(2):153-61.

45. Moser DK, Heo S, Lee KS, Hammash M, Riegel B, Lennie TA, et al. 'It could be worse ... lot's worse!' Why health-related quality of life is better in older compared with younger individuals with heart failure. Age Ageing. 2013;42(5):626-32.

46. Lewis EF, Lamas GA, O'Meara E, Granger CB, Dunlap ME, McKelvie RS, et al. Characterization of health-related quality of life in heart failure patients with preserved versus low ejection fraction in CHARM. Eur J Heart Fail. 2007 Jan;9(1):83-91.

47. Hoekstra T, Jaarsma T, Van Veldhuisen DJ, Hillege HL, Sanderman R, Lesman-Leegte I. Quality of life and survival in patients with heart failure. European Journal of Heart Failure. 2013;15(1):94-102.

48. Hou N, Chui MA, Eckert GJ, Oldridge NB, Murray MD, Bennett SJ. Relationship of age and sex to health-related quality of life in patients with heart failure. Am J Crit Care. 2004 Mar;13(2):153-61.

49. Davis LL. A Qualitative Study of Symptom Experiences of Women With Acute Coronary Syndrome. J Cardiovasc Nurs. 2017 Sep/Oct;32(5):488-495. doi: 10.1097/JCN.0000000000000381. PMID: 27870722; PMCID: PMC5436957.

50. Lichtman JH, Leifheit EC, Safdar B, Bao H, Krumholz HM, Lorenze NP, Daneshvar M, Spertus JA, D'Onofrio G. Sex Differences in the Presentation and Perception of Symptoms Among Young Patients With Myocardial Infarction: Evidence from the VIRGO Study (Variation in Recovery: Role of Gender on Outcomes of Young AMI Patients). Circulation. 2018 Feb 20;137(8):781-790. doi: 10.1161/CIRCULATIONAHA.117.031650.

51. Cajita MI, Cajita TR, Han HR. Health Literacy and Heart Failure: A Systematic Review. J Cardiovasc Nurs. 2016 Mar-Apr;31(2):121-30. doi: 10.1097/JCN.0000000000000229. PMID: 25569150; PMCID: PMC4577469

52. Cajita MI, Hodgson NA, Lam KW, Yoo S, Han HR. Facilitators of and Barriers to mHealth Adoption in Older Adults With Heart Failure. Comput Inform Nurs. 2018 Aug;36(8):376-382. doi: 10.1097/CIN.0000000000000442. PMID: 29742549; PMCID: PMC6086749.

53. Bowling A. Just one question: If one question works, why ask several? J Epidemiol Community Health. 2005;59(5):342-5.

54. Lawson C, Crothers H, Remsing S, Squire I, Zaccardi F, Davies M, Bernhardt L, Reeves K, Lilford R, Khunti K. Trends in 30-day readmissions following hospitalisation for heart failure by sex, socioeconomic status and ethnicity. EClinicalMedicine. 2021 Jul 14;38:101008. doi: 10.1016/j.eclinm.2021.101008. PMID: 34308315; PMCID: PMC8283308.
